# Histological Grading of Hepatocellular Carcinoma—A Systematic Review of Literature

**DOI:** 10.3389/fmed.2017.00193

**Published:** 2017-11-10

**Authors:** Sebastiao N. Martins-Filho, Caterina Paiva, Raymundo Soares Azevedo, Venancio Avancini Ferreira Alves

**Affiliations:** ^1^Departamento de Patologia, Faculdade de Medicina FMUSP, Universidade de São Paulo, São Paulo, Brazil; ^2^Laboratorio de Patologia do Fígado LIM 14, Hospital das Clinicas HCFMUSP, Faculdade de Medicina, Universidade de São Paulo, São Paulo, Brazil

**Keywords:** hepatocellular carcinoma, histological grading, grading systems, Edmondson and Steiner, prognosis

## Abstract

**Background:**

Histological grading typically reflects the biological behavior of solid tumors, thus providing valuable prognostic information. This is also expected in hepatocellular carcinoma (HCC), although limited access to biopsy samples and a lack of standardization might hinder its full predictive value in this cancer.

**Objectives:**

In order to better understand the current practices of histological grading in HCC, we examined the latest publications addressing its impact on the outcome of patients following surgical treatment.

**Methods:**

We searched the PubMed (MEDLINE) database under the headings “hepatocellular carcinoma,” “grade OR grading,” and “prognosis.” Qualitative and quantitative assessment of publications was performed according to the reference they used to grade their tumors (e.g., Edmondson–Steiner, World Health Organization).

**Results:**

We reviewed a total of 216 articles: 114 enclosed adequate information and were included herein. Among these, we found divergences and inaccuracies in the histological grade assessment of this cancer, which might have led to a non-standardized grade distribution, with further impact on data analysis. Nevertheless, in most of them, poor tumor differentiation correlated with worse prognosis, expressed by lower overall and/or disease-free survival.

**Conclusion:**

While histological grading of HCC has an important prognostic role, there is an unsatisfactory heterogeneity on the microscopic assessment of this tumor, urging for a movement toward standardization.

## Introduction

Image-guided needle biopsies and histopathological evaluation are the gold standard for the diagnosis of most solid organ neoplasms. They also allow for tumor subtyping and pave the way for integrated studies in cellular and molecular biology that will ultimately improve the management of patients with cancer. However, considering the current clinical guidelines, needle biopsies are seldom required for hepatocellular carcinoma (HCC) diagnosis, being reserved for suspicious, but non-diagnostic lesions on imaging examinations (1, 2). This remarkable discrepancy to the general oncological practice restricts our ability to define and select subgroups of patients for new drugs and clinical trials, and might explain the scarcity of effective therapeutic strategies in this cancer (3–5).

On the other hand, the gross and histological evaluation of HCC specimens obtained by surgical resection has continuously allowed for the identification of histological subtypes including fibrolamellar (6, 7), lymphoepithelioma-like (8), and steatohepatitic HCC (9), as well as morphomolecular features such as the distinct patterns of vascular invasion (10) and the expression of stemness markers such as Keratin 19 (assessed by immunohistochemistry or by molecular pathology) (11, 12), well-established independent prognostic factors in HCC.

A major prognostic feature in solid tumors from virtually every organ, histologic grading is also expected to reflect the tumor’s biological behavior in HCC. However, the classical and most commonly adopted grading system for this cancer is Edmondson–Steiner (ES), published in the far 1954 (13), which might need to be revalidated or even updated according to more contemporary histopathological approaches.

To better understand the current practices of histological grading in HCC, we examined the latest publications addressing their impact on survival and recurrence in patients following surgical treatment. Strikingly, we found a great divergence regarding histological grade assessment in this cancer. Herein, we present these findings, as we briefly review some of the grading systems for HCC and discuss a potential approach for a higher consonance on the microscopic assessment of this tumor.

## Methods

On August 3, 2016, we searched the PubMed (MEDLINE) database to raise potentially relevant articles. Keywords were “hepatocellular carcinoma,” “grade OR grading,” and “prognosis” appearing on the title or abstract. We selected all the publications from January 1, 2011, to August 3, 2016, and limited the search to include only those available in full text, in English, and with humans as the species under the study. We excluded the reviews, those with irrelevant content, repeated or inconsistent data, and those in which the final intervention was not liver resection (LR), nor liver transplantation (LT).

The information collected from each article included first author name, year of publication, interval of data collection, modality of surgical treatment, previous interventions, number of samples, histological grading system, and its impact on outcome (univariate and multivariate analyses, when available) (Table S1 in Supplementary Material).

The studies were initially classified based on the modality of surgical treatment employed: LT, LR, and LR + LT. Considering that the clinical management of patients varies considerably following LR and LT, we conducted the descriptive analysis separately for these groups, and excluded the articles that had dealt with both interventions.

We then screened for the reference (depicted on the methods or bibliography) each publication used to grade their tumors. Studies that referred to the ES 1954 publication were analyzed altogether (ES subgroup). Studies that have referred the World Health Organization (WHO) book on the “Classification of Tumours of the Digestive System” as their main reference were considered, in our analysis, a different subgroup (WHO subgroup). We also identified additional histological classification/references (aggregated as “OTHERS”) and studies that did not inform which grading system they have used to analyze HCC (NI subgroup).

Finally, we selected the studies from the ES and WHO subgroups that have disclosed the univariate impact of the histological grade with a 95% confidence interval (CI), and organized forest plots to quantify the importance of histological grading in HCC. Different estimates of relative risk (odds ratio and hazard ratio) were combined, as previously described (14). Fixed and random effects meta-analyses and forest plot-based estimates for hazard ratios were calculated by inverse variance weighting using the R Project for Statistical Computing (R Core Team, 2016), with R Commander package (version 2.3, October 2016) and plugin EZR (version 1.33, September 2016). Eligible studies that performed their analyses in two different cohorts had both results included. Due to the limited number of articles that analyzed data following LT, we restrained our quantitative evaluation to the LR publications. Additional graphs were designed with the software package SPSS 22.0 (SPSS, Inc., Chicago, IL, USA). In all situations, a *p* < 0.05 was considered significant.

## Results

### Characterization of HCC Histological Grading in the Literature

**Figure 1:**
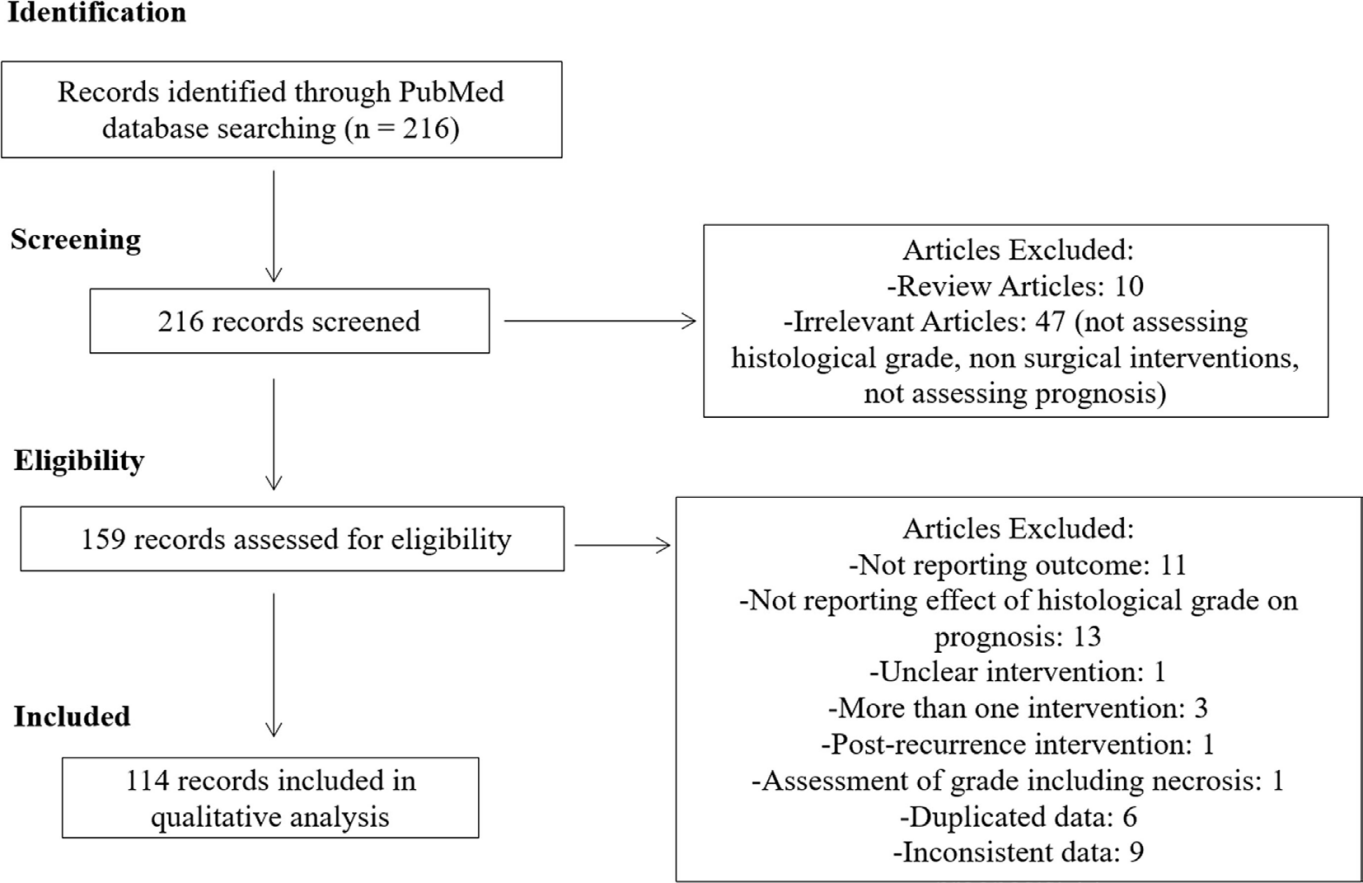
Flow diagram of the study selection process.

We identified 216 articles in our online database search. After screening and assessing our eligibility criteria, 114 studies were selected and thoroughly analyzed. A summary of our study selection process is summarized in Figure 1.

**Figure 2:**
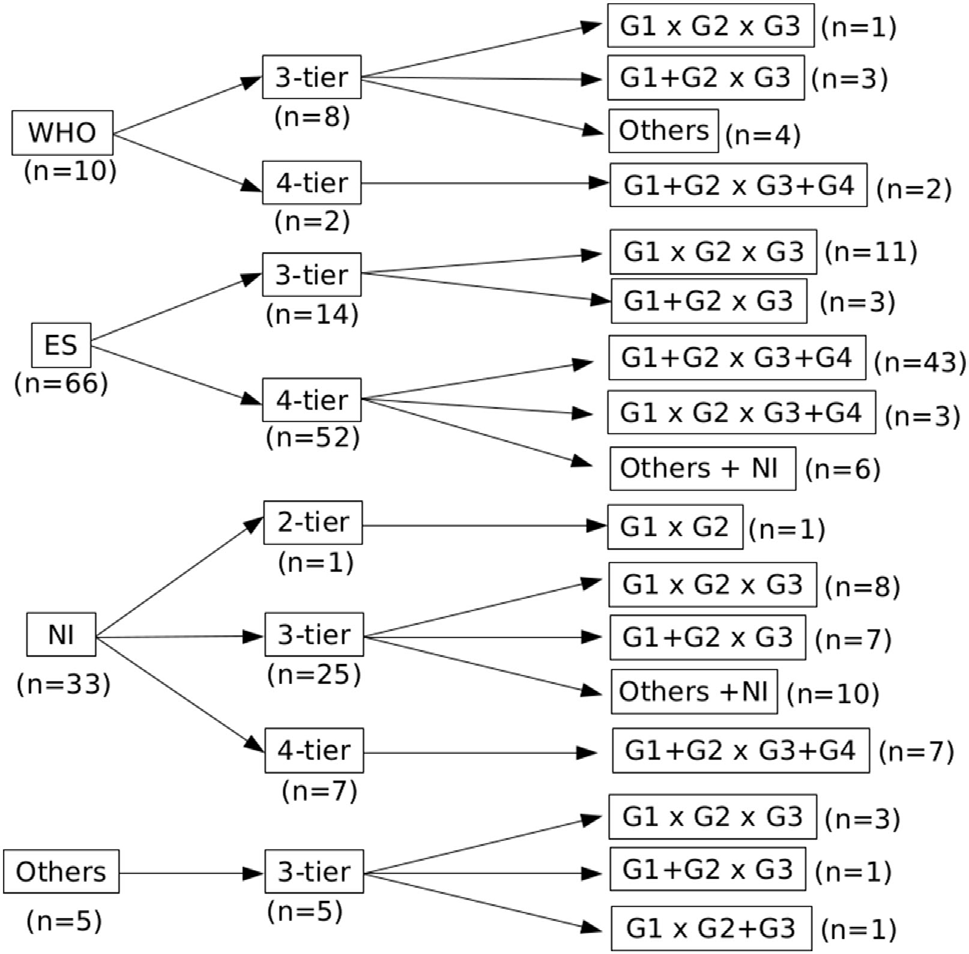
Distribution of the studies according to the grading reference, number of tiers, and data analysis. There is a lack of consensus on the classification of hepatocellular carcinoma in the literature, illustrated by different grading systems and number of tiers.

Most of the studies included in our analysis belonged to the ES subgroup (*n* = 66) and prioritized a 4-tier histological grade distribution. In contrast, and most likely due to differences from the ES classification, WHO (*n* = 10) and OTHERS (*n* = 5) reference subgroups organized tumors in 3-tiers (Figure 2). In this latter subgroup, we identified four publications that used the histological classification proposed by the Union for International Cancer Control (*sic*) and one that used the classification from the Liver Cancer Study Group of Japan.

**Figure 3:**
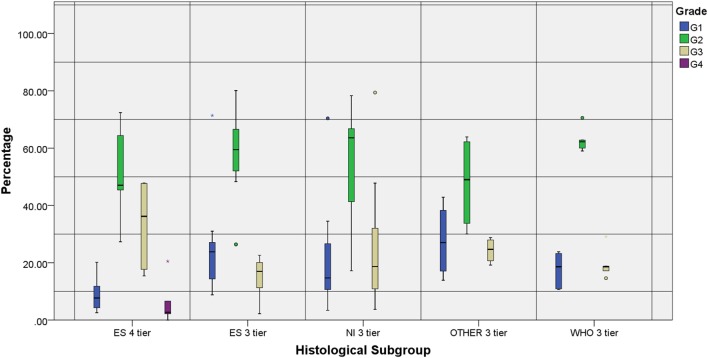
Individual grade distribution according to the reference subgroup and number of tiers. All groups represented show a higher distribution of the intermediate grades compared to the marginal ones. Two studies that were not included in the chart—WHO 4-tier (G1: 8.9%, G2: 45.5%, G3: 42.7% and G4: 2.8%) and NI 4-tier (G1: 7%, G2: 59%, G3: 32% and G4: 2%)—reproduced the distribution observed in the ES 4-tier category.

The number of tiers also showed some disparities: authors under the ES subgroup who organized tumors in 3-tiers had a significantly higher percentage of G1 and lower percentage of G3 tumors when compared to ES 4-tier, while a more identical distribution when compared to the other 3-tier subgroups (Figure 3).

The reference and particularly the number of tiers also played an important role on how the histological grades were organized prior to data analysis (Figure 2). While 3-tier studies tended to assess each grade individually (G1 × G2 × G3), 4-tier studies usually dichotomized them in low (G1 + G2) and high grades (G3 + G4); some of them even presented their results with different grades combined.

Interestingly, large-scale clinical and genomic data constantly support this latter approach, suggesting that the biological behavior of G2 HCC is closer to G1 than to G3 (15, 16). Once again, we analyzed the distribution of low grade (G1 and G2) and high grade (G3 and, when available, G4) HCC: overall, 38.0% of the tumors were considered high grade. However, there were only 27.8% of high-grade tumors in the WHO subgroup, as opposed to 38.8 and 39.8% in the ES and NI subgroups, respectively.

### Impact of the Histological Grade in the Prognosis of HCC Patients Treated with LT

Twelve cohorts—distributed in 11 studies—evaluated the impact of the histological grade on the prognosis of HCC patients submitted to LT. Results for the univariate analysis were displayed in eight cohorts: correlation between higher grades and poor outcome was observed in 5 (62.5%) of them. Ten cohorts displayed the results for the multivariate analysis and 6 (60.0%) found correlation between outcome and grade.

Only four studies clearly described their grading classification: all belonged to the ES subgroup. The two studies that organized tumors in 4-tiers found correlation between grade and outcome in both univariate and multivariate analyses. The two remaining publications organized tumors in 3-tiers: one found correlation in the univariate, but not in the multivariate analysis, and the other showed only a trend between grade and outcome, although not statistically significant.

### Impact of the Histological Grade in the Prognosis of HCC Patients Treated with LR

From the 103 studies based on LR, 86 had performed univariate analysis including histological grade. Overall, 56 (65.1%) of these showed a better outcome for patients with a lower histological grade. One study that divided patients into two categories—“AFP negative” (≤20 ng/ml) and “AFP positive” (>20 ng/ml)—found that grading was a significant predictor of survival only in this latter category (17). One article assessed patients with single small (<2 cm) and single large (>2 cm) HCC separately. Favorable overall survival for patients with better differentiated tumors was found for larger but not for smaller HCC (single large HCC G1 + G2: 71.63%, G3 + G4: 28.37%/single small HCC G1 + G2: 81.08%, G3 + G4: 18.92%) (18). Nonetheless, another study including only tumors ≤2 cm indicated significantly lower overall recurrence, advanced recurrence within 1 year, and advanced recurrence within 2 years in better differentiated HCC (19).

Among the 77 articles that declared the grading system used, 64 assessed prognostic significance of tumor grade in the univariate level. Forty three (67.2%) found significant correlation between grading and prognosis. For the most commonly adopted grading system (ES 4-tier) histological grade was a significant predictor of outcome in 32 of 42 (76.2%) articles. Among the eight studies that used ES as a 3-tier system, 5 (62.5%) showed significant correlation. In the WHO subgroup, lower grade was associated with better outcome in only 2 (25%) of the eight studies and 1 (50%) of the two studies when it was considered a 3- and 4-tier classification, respectively. Of note, one study within WHO 3-tier compared “non-poor” tumors (NP: containing only G1 and G2), “poorly containing” (PC: containing G3, predominant G1 or G2), and poorly differentiated (PD: predominant G3). Significantly better overall survival and recurrence-free survival were found for NP when compared to PC and to PD, whereas no significant difference was detected between PC and PD cases (20). In a similar fashion, an ES 4-tier study demonstrated that tumors with focal areas of G3 have worse outcome when compared to homogeneous G2 tumors (15). For the subgroup “OTHERS,” univariate analysis of the impact of grading in prognosis showed correlation in 3 (75%) of the four studies.

**Figure 4:**
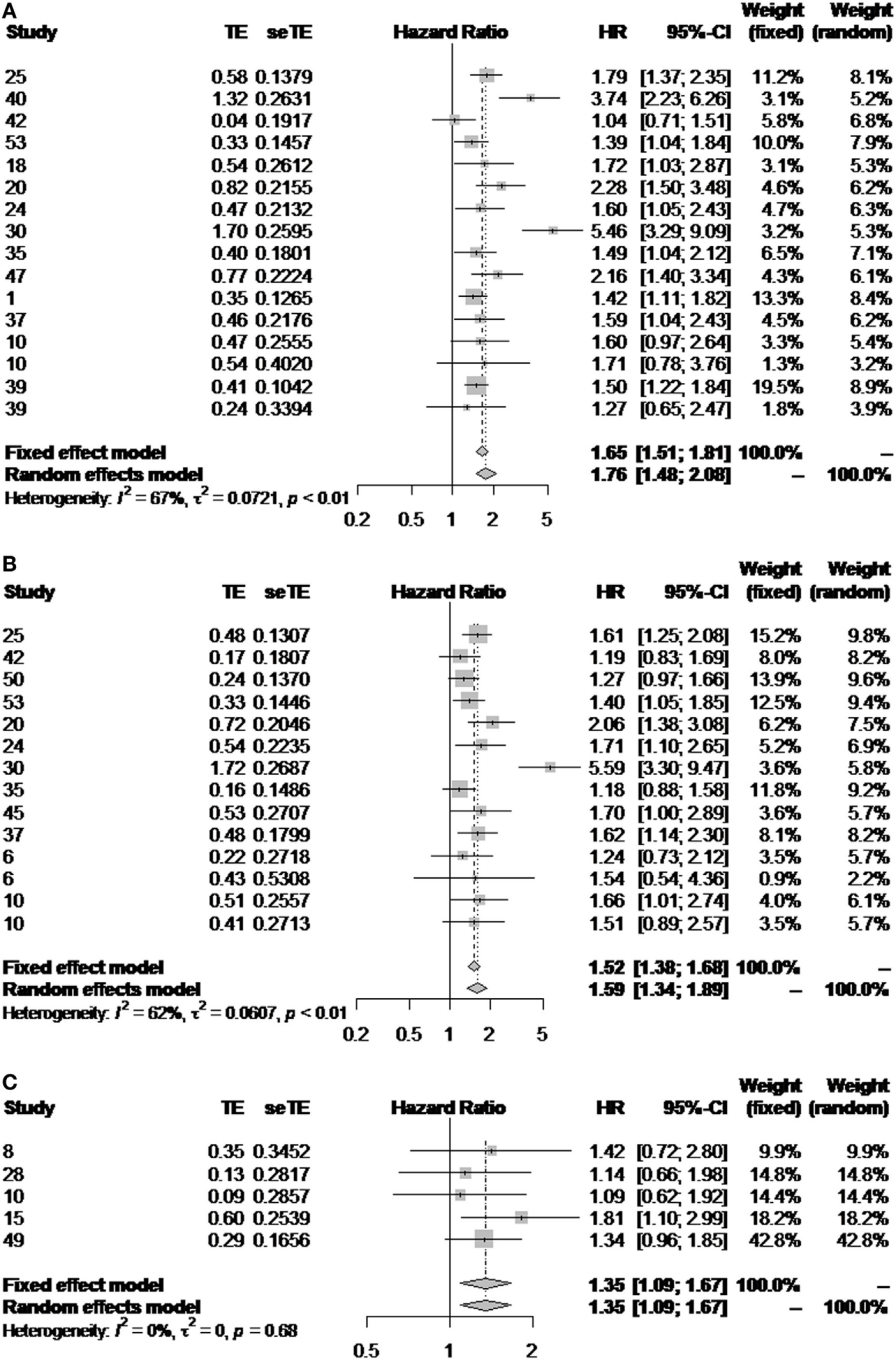
Impact of the histological grade in outcome. Forest plot diagrams illustrating the impact of the ES classification on overall **(A)** and disease-free survival **(B)**, and the impact of the WHO classification on overall survival **(C)**.

When results are pooled, histological grade shows correlation with survival for both ES and WHO subgroups. In the former, however, we observed a high heterogeneity (*I*^2^ > 60%, *p* < 0.01), either when evaluating the impact of grade on overall or disease-free survival (Figures 4A,B). Interestingly, there was a higher consistency in the results from the WHO subgroup (Figure 4C). In this latter subgroup, however, analysis was restrained to overall survival due to the limited number of publications evaluating its impact on disease-free survival.

## Discussion

This comprehensive review of recent international literature on HCC grading demonstrates that, still now, the most broadly adopted reference for histological grading in HCC is the ES system, published in *Cancer* in 1954 (13). The WHO, on its prestigious “blue book” series, displays an adaptation of the original reference on its “WHO Classification of Tumours of the Digestive System,” whose latest edition date from 2010 (21). Despite being published more than 60 years ago, the original classification is transcribed and still recommended by the College of American Pathologists on its protocol for examination of HCC, updated in 2011 (22).

Both the 1954 publication and the WHO 2010 book share a lot of similarities in their characterizations, but do not completely overlap, especially if the morphologist strictly follows their descriptions. Among these similarities are the facts that both recognize four different grades for HCC, and consider a combination of structural and cellular features for defining the final grade. Some differences encompass the mild cytological atypia and acinar architecture, which can accompany the thin trabecular tumors that falls under WHO’s grade I (well-differentiated) HCC, but can also be described by the “marked resemblance to normal hepatic cells” and frequent acini, now in ES’s grade II (Figure S1 in Supplementary Material). In fact, Edmondson and Steiner even state that “Grade I is best reserved for those areas in Grade-II carcinomas where the difference between the tumor cells and hyperplastic liver cells is so minor that diagnosis of carcinoma rest upon the demonstration of more aggressive growth in other parts of the neoplasm.” This description seems more illustrative of HCC in which the differentiation from dysplastic nodules or adenomas is challenging and relies on the evaluation of other areas of the tumor.

Furthermore, defining WHO’s worst grade as undifferentiated is potentially misleading, as this pathological terminology is reserved for anaplastic tumors in which the embryonal lineage is yet to be established. To avoid further confusion, we defend the use of “undifferentiated HCC” for PD tumors with focal anaplastic areas and the use of “undifferentiated carcinoma” for homogenously anaplastic cancer following immunohistochemical demonstration of epithelial markers, yet no characterization of hepatocellular lineage. Additional differences and exempts from WHO and ES can be found in Table 1.

**Table 1:** Histological features from Edmondson and Steiner (ES) publication and WHO book.

Reference	Grades	Architecture	Cytology	Other features
World Health Organization (21)	Well differentiated	Thin trabecular, frequent acinar structures	Minimal atypia	Fatty change is frequent
	Moderately differentiated	Trabecular (3 or more cells in thickness) and acinar	Abundant eosinophilic cytoplasm, round nuclei with distinct nucleoli	Bile or proteinaceous fluid within acini
	Poorly differentiated	Solid	Moderate to marked pleomorphism	Absence of sinusoid-like blood spaces
	Undifferentiated	Solid	Little cytoplasm, spindle, or round-shaped cells	—

Edmondson and Steiner (13)	Grade I	—	—	Areas of carcinoma where distinction from hyperplastic liver is difficult
	Grade II	Trabecular, frequent acini (lumen varying from tiny canaliculi to large thyroid-like spaces)	Resemblance to normal hepatic cells; larger nuclei; abundant acidophilic cytoplasm	Cell borders sharp and clear cut; acini containing bile or protein precipitate
	Grade III	Distortion of trabecular structure, acini less frequent than grade II	Larger, more hyperchromatic nuclei, granular but less acidophilic cytoplasm	Acini are less frequent; tumor giant cells may be numerous
	Grade IV	Medullary, less trabeculae, rare acini	Highly hyperchromatic nuclei, scanty cytoplasm, with fewer granules	Loss of cell cohesiveness; giant, spindle or short-plump cells can be found

Besides subtle, these nuances seem to have induced several authors to classify HCC in 3-tiers when referring to the WHO (Figure 2), and provide the basis for the lower percentage of high-grade tumors when comparing WHO (24.6%) and ES (37.0%). This, in turn, might account for the differences regarding outcome in these subgroups, since the distinction between G2 and G3 seems to be the cornerstone for histological grade impact on outcome. In fact, while 73.5% of the publications in the ES subgroup following LR found a statistically significant correlation between grade and outcome, only 30% in the WHO subgroup did so.

On the other hand, authors who use the WHO as their reference tend to grade their tumors more homogeneously, thus presenting less-conflicting results (Figures 2 and 4C). For instance, the percentage of G1 tumors ranges from 8.9 to 23.8% when the WHO is the reference, and goes from 2.38 to 78% in the ES subgroup (including ES 3-tier and 4-tier). These results are intriguing, especially considering that the original classification restrains the diagnosis of G1 tumors and recommends HCC to be classified according to the worst area. Variability in tumor characteristics between the centers could explain such divergences, though it is legitimate to raise the concern that this is partially induced by different interpretations of the ES classification, thus affecting the way of grading.

On top of those reproducibility issues, the ES grading classification was proposed on an autopsy cohort (further limiting its predictive value) and, at that time, could not incorporate the distinct HCC histological patterns and clinical and molecular advances. It is important to acknowledge that long-standing and iconic classifications of neoplasms from other organs such as the Gleason System for prostate carcinomas (pivotal for its visual guide and for the assessment of phenotypical tumor heterogeneity) and the SBR classification for breast carcinomas (clearly defining specific criteria—architecture, nuclear atypia, and mitoses) have been challenged by the molecular characterization of these tumors. The remarkable advances achieved in the organs where pathology has remained the core of medical approach for the diagnosis have yielded a new paradigm of “morpho-molecular classifications,” leading to fantastic improvements on the clinical management of these tumors (23).

Attempts for improving grade assessment of HCC were made. For instance, Goodman and Ishak, in the second edition of the AFIP Liver Fascicles, proposed a modified ES grading system, placing bigger emphasis on nuclear pleomorphism. While pure grade I tumors were still unlikely and undistinguishable from adenomas, differences would encompass the following grades (24). Noticeably, giant cell carcinomas were shifted from G3 in the original classification to G4 in this modified version (Figure S2 in Supplementary Material).

Similarly, Lauwers et al. described a histologic predictive index for HCC, combining nuclear features and microvascular invasion to stratify tumors in fair and poor prognosis (25). Although promising, these classifications were not validated by other groups/bigger cohorts and are not recommended by current protocols.

**Figure 5:**
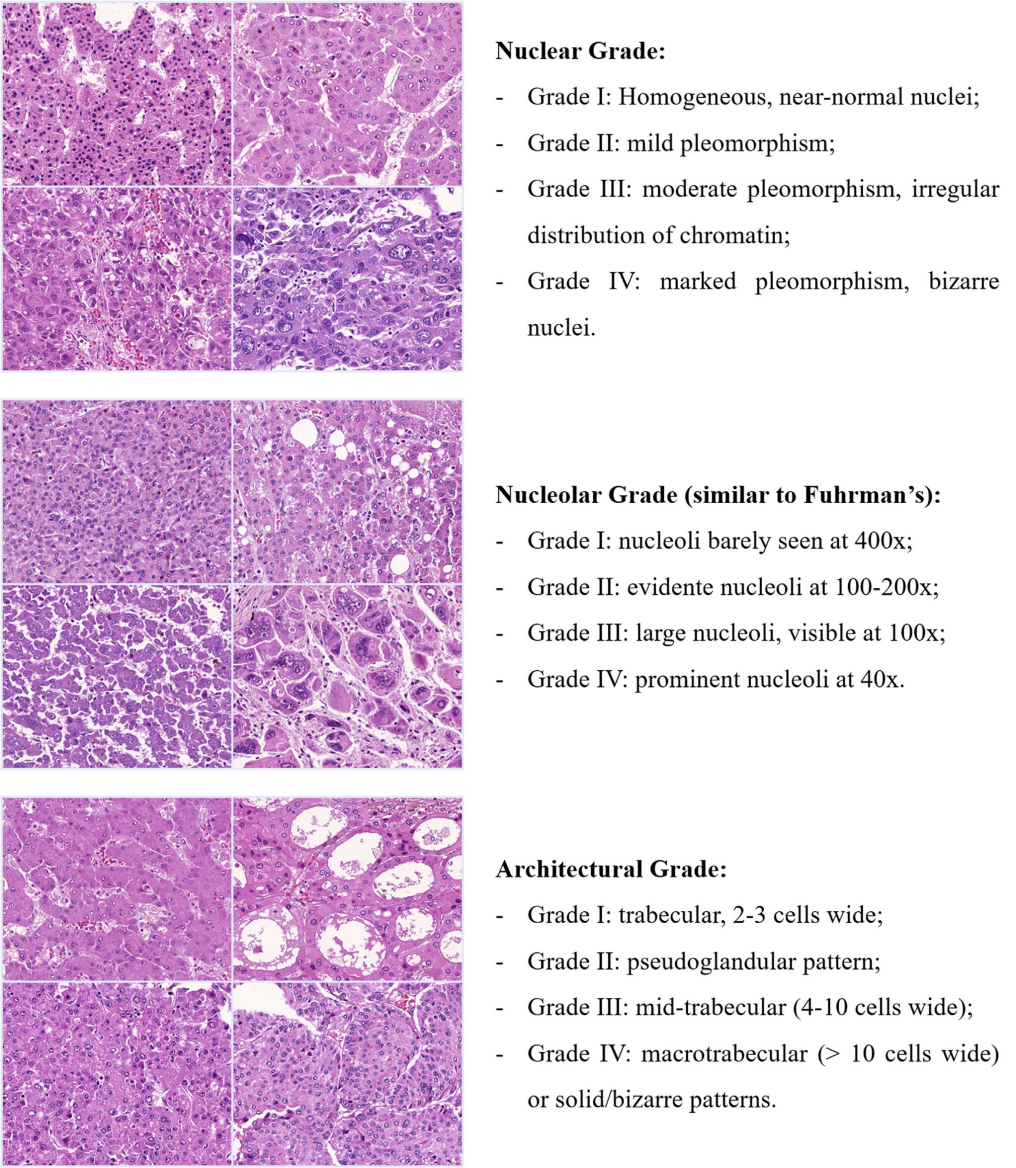
Potential approach for a new grading classification on HCC. Tumors would be classified into four grades for each histological feature and, depending on the combined scored, stratified in low or high grade. Each feature or their combination would then be cross-examined with the patterns of vascular invasion (micro and macrovascular), the expression of stem-like markers (e.g., Keratin 19) and even with the HCC molecular subclasses. Exemplified here are nuclear, nucleolar, and architectural grade, but other histological variables such as cellularity and even mitotic index could also be explored.

There is thus a fertile ground for an update or even a new grading classification for HCC. Considering the aforementioned examples on prostate and breast carcinoma, HCC should also be classified according to more objective criteria, acquiescent to outcome, histological patterns, and molecular subclasses. A potential approach would be to individually classify different histological parameters (such as architecture, cellularity, nuclear and nucleolar pleomorphism, and, perhaps, mitoses/proliferation index), which, desirably, should be scored to yield the stratification of tumors in low or high grade (Figure 5).

Even with great variability and lack of consensus, grading appears to be a relevant prognostic factor in HCC: in most of the studies, poor tumor differentiation correlated with worse prognosis, expressed by lower overall and/or disease-free survival.

While we could not address regional differences, we acknowledge them as possible confounders to our results. Nevertheless, for the main objective of assessing histological grade in HCC outcome, we tried to overcome that limitation by pooling different studies together. Also, it would have been ideal to compare the differences between the ES and WHO references in the same cohort to better understand how their divergences really affect histological grading and its impact in outcome. However, and considering that those divergences are subtle, a blinded approach and a huge cohort would be required.

## Conclusion

The present comprehensive review of the literature from 2011 to 2016 clearly shows that histological grading of HCC can still be considered a relevant prognostic marker. Our view is that, in the constant search for predictors of a favorable response to potentially curative treatments, histological grade might be an important variable, to which other biomarkers can sum and help determine prognosis. However, to be able to assess its authentic value, better definitions and greater uniformity are needed.

## Author Contributions

SNMF and CP contributed equally to this work. SNMF worked on data analysis and interpretation, and on the drafting of the article. CP worked on data collection and on the drafting of the article. RSA helped with data analysis. VAFA performed a critical review of the article.

## Conflict of Interest Statement

The authors declare that the research was conducted in the absence of any commercial or financial relationships that could be construed as a potential conflict of interest.

## Supplementary Material

Figure S1 Histological representation of a low-grade hepatocellular carcinoma. Hepatocellular carcinoma with trabecular architecture (three to four cell thickness) and mild cytological atypia. Some acini can be observed, filled with proteinaceous fluid. This tumor is classified as well differentiated according to the WHO criteria, but is better aligned under ES’s grade II tumors.

Figure S2 Histological representation of a high-grade hepatocellular carcinoma. This tumor would be classified as G3 according to ES, but as G4 according to the modified histologic classification proposed by Goodman and Ishak. Despite the bizarre-looking cells, this tumor retained the immunohistochemical expression of HepPar 1.

Table S1 HCC-related articles evaluating the impact of histological grading on outcome
