# Supplementary figures and images for: Histological Grading of Hepatocellular Carcinoma—A Systematic Review of Literature
Source: Front Med (Lausanne). 2017 Nov 10;4:193. doi: 10.3389/fmed.2017.00193 (PMC5701623; doi:10.3389/fmed.2017.00193)

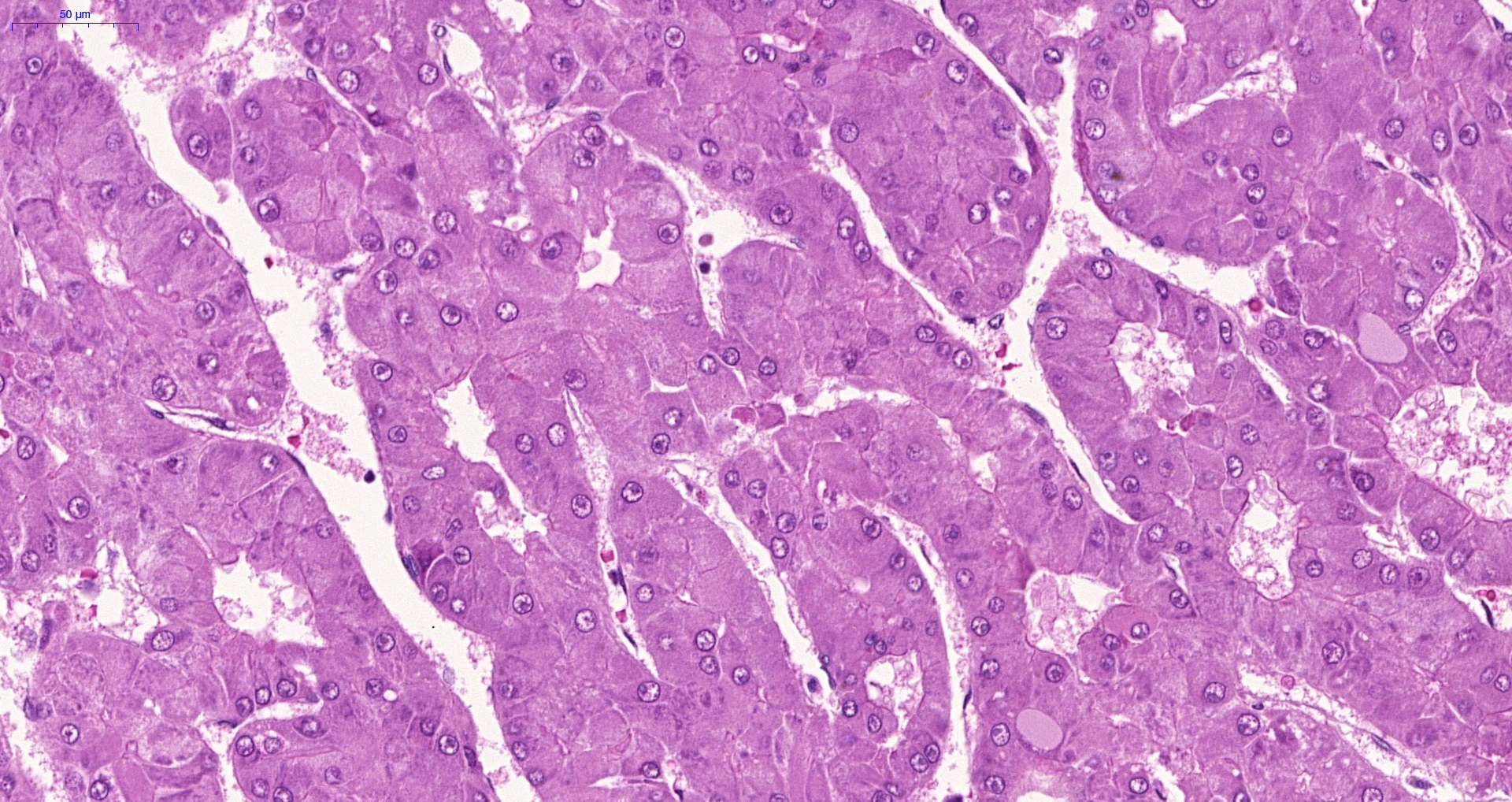

Supplement: Figure S1 — Histological representation of a low-grade hepatocellular carcinoma. Hepatocellular carcinoma with trabecular architecture (three to four cell thickness) and mild cytological atypia. Some acini can be observed, filled with proteinaceous fluid. This tumor is classified as well differentiated according to the WHO criteria, but is better aligned under ES’s grade II tumors. [file Image_1.TIF]

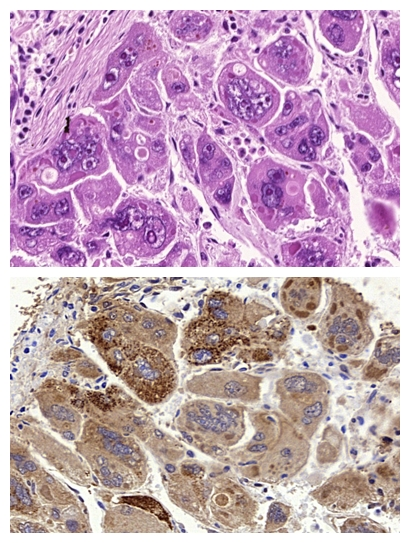

Supplement: Figure S2 — Histological representation of a high-grade hepatocellular carcinoma. This tumor would be classified as G3 according to ES, but as G4 according to the modified histologic classification proposed by Goodman and Ishak. Despite the bizarre-looking cells, this tumor retained the immunohistochemical expression of HepPar 1. [file Image_2.TIF]
