# Supplementary material for: Histological Grading of Hepatocellular Carcinoma—A Systematic Review of Literature
Source: Front Med (Lausanne). 2017 Nov 10;4:193. doi: 10.3389/fmed.2017.00193 (PMC5701623; doi:10.3389/fmed.2017.00193)
Supplement: Table S1 — HCC-related articles evaluating the impact of histological grading on outcome [file Table_1.DOCX]

Supplementary Table 1 – HCC-related articles evaluating the impact of histological grading on outcome.

| **Author** | **Year** | **Interval of data collection** | **Intervention** | **Pre-intervention**  **Combined therapy** | **n** | **Grading Reference** | **Tiers** | **Data Analysis** | **Univariate analysis** | **Multivariate analysis** |
| --- | --- | --- | --- | --- | --- | --- | --- | --- | --- | --- |
| Shamsaeefar et al(1) | 2015 | January 2008-December 2013 | LT | TACE (3.4%), RFA (2.3%) | 88 | NI | 3-tier | NI | NI | TFS: OR: 14.42 (95%CI: 3.652-56.95), P<0.001 |
| Andreou et al(2) | 2014 | 1989-2010 | LT | TACE (27%) | 364 | NI | 3-tier | G1+G2 x G3 | OS: median survival (months) 125 vs 51, P<0.0001 | NS |
| Park et al(3) | 2014 | February 1999-October 2010 | LT | TACE (65.4%) | 205 | ES | 4-tier | G1+G2 x G3+G4 | NI | Recurrence: HR=2.249 (95%CI: 1.194-4.234), P=0.012 |
| Iguchi et al(4) | 2014 | July 1999-February 2011 | LT | NI | 141 | NI | 3-tier | G1+G2 x G3 | NI | RFS: HR=1.731 (95%CI: 0.578‐5.189), P=0.327 |
|  |  |  |  |  | 61 |  |  |  |  | RFS: HR=1.423 (95%CI: 0.743‐2.728), P=0.287 |
| Hoyos et al(5) | 2015 | April 1 2004-May 31 2013 | LT | NI | 54 | NI | 3-tier | G1 x G2+G3 | Recurrence: P=0.3 Survival: 68 vs 52.7 months, P=0.3 | not included |
| Wang et al(6) | 2015 | 2008-2013 | LT | previous treatments (42%) | 238 | NI | 3-tier | G1 x G2 x G3 | Survival: HR=1.796 (95%CI: 1.228-2.625), P=0.003 | Survival: β=0.437, P=0.023 |
| Varona et al(7) | 2015 | April 1996-December 2012 | LT | previous treatment (52.3%) | 91 | ES | 3-tier | G1 x G2 x G3 | Recurrence: 50% vs 12% vs 6%, P=0.14 | - |
| Cui et al(8) | 2015 | 2002-2011 | LT | NI | 104 | ES | 4-tier | G1 x G2 x G3 (no G4) | 5-year survival: 76.2% vs 43.3% vs 25%, P<0.001 DFS: 71.4% vs 40.3% vs 18.8%, P<0.001 | 5-year survival: OR=0.422 (95%CI: 0.235-0.759), P=0.004 DFS: OS=0.498 (95%CI: 0.282-0.878), P=0.016 |
| Wang et al(9) | 2015 | July 2007-August 2012 | LT | NI | 76 | NI | 3-tier | G1+G2 x G3 | Recurrence: recurrence group: 83.9% differentiated / non-recurrence group: 95.6% differentiated, P=0.114 RFS: 47.95 months [40.22–55.68] vs 26.88 months [13.12–40.62], P=0.6 OS: 49.60 months (42.44–56.75) vs 33.19 months (42.16–55.89), P=0.781 | Recurrence: OR=10.412 (95%CI: 1.475–73.498), P=0.019 RFS: HR=4.114 (95%CI: 1.032–16.391), P=0.045 |
| An et al(10) | 2012 | August 2000-July 2010 | LT | TACL (80%), other treatments | 85 | NI | 3-tier | G1 x G2+G3 | OS: P=0.02 RFS: P=0.03 | OS: HR=1.28 (95%CI: 0.46-3.54), P=0.63 RFS: HR=1.01 (95%CI: 0.26-3.87), P=0.91 |
| Wan et al(11) | 2014 | January 2007-December 2010 | LT | LR, TACE, RFA, PEI, gamma knife (50%) | 130 | ES | 3-tier | G1+G2 x G3 | OS (5-year): 46.5% vs 24.9%, P=0.026 RFS (5-year): 44.1% vs 30.9%, P=0.043 | NS |
| Hua et al(12) | 2015 | June 2006-July 2012 | LR | no chemotherapy, radiotherapy or immunotherapy | 92 | NI | 2-tier | OTHER | OS: HR=2.718 (95%CI: 1.336-5.257), P=0.007 | OS: HR=2.407 (95%CI: 1.262-4.832), P=0.012 |
| Zhu et al(13) | 2015 | 2004-2005 | LR | NI | 58 | NI | 3-tier | G1 x G2 x G3 | Survival: P=0.001 | Survival: HR=4.924 (95%CI: 1.985–12.218), P=0.001 |
| Gu et al(14) | 2014 | 2003-2010 | LR | no chemotherapy, radiotherapy or immunotherapy | 100 | NI | 3-tier | G1+G2 x G3 | OS: HR=0.44 (95%CI: 0.245–0.783), P=0.005 DFS: HR=0.45 (95%CI: 0.252–0.807), P=0.007 | OS: HR=0.74 (95%CI: 0.384–1.438), P=0.378 DFS: HR=0.79 (95%CI: 0.425–1.467), P=0.455 |
| Kishida et al(15) | 2015 | 2005-2010 | LR | previous treatments (29.8%) | 104 | NI | 3-tier | G1 x G2+G3 | RFS: HR=1.61 (95%CI: 0.69–3.73), P=0.27 | NI |
| Chen et al(16) | 2015 | 2007-2012 | LR | no chemotherapy, radiotherapy or local treatments | 75 | NI | 3-tier | G1+G2 x G3 | OS: HR=1.263 (95%CI: 0.887–1.798), P=0.196 DFS HR=1.193 (95%CI: 0.853–1.667), P=0.303 | not included |
| An et al(17) | 2015 | January 2006-December 2011 | LR | preoperative TACE (88.4%) | 251 | NI | 3-tier | G1 x G2 x G3 | OS: HR=2.234 (95%CI:1.535-3.253), P<0.001 RFS: HR=1.770 (95%CI: 1.316-2.381), P<0.001 | OS: HR=1.701 (95%CI: 1.116-2.594), P=0.014 RFS: HR=1.487 (95%CI: 1.075-2.059), P=0.017 |
| Lu et al(18) | 2015 | 2004-2007 | LR | no prior treatment | 164 | NI | 3-tier | G1 x G2+G3 | OS: RR=1.057 (95%CI: 0.709-1.576), P=0.786 | OS: RR=0.784 (95%CI: 0.517-1.186), P=0.249 |
| Zhang et al(19) | 2012 | January 2001-October 2008 | LR | No previous treatment | 400 | NI | 3-tier | G1 x G2 x G3 | NS | NS |
| Faber et al(20) | 2014 | January 2000-September 2010 | LR | NI | 141 | NI | 3-tier | G1 x G2 x G3 | CS: exp(B)=1.034 (0.787-1.360), P=0.81 RFS: exp(B)=1.070 (0.824-1.389), P=0.61 | NS/not included |
| Ni et al(21) | 2013 | May 2002-January 2005 | LR | No previous chemotherapy | 65 | NI | 3-tier | G1+G2 x G3 | OS: χ²=19.293, P<0.001 | OS: HR=2.016 (95%CI:1.157-3.512), P=0.013 |
| Wei et al(22) | 2012 | 2001-2007 | LR | No previous chemotherapy or chemoembolization | 91 | NI | 3-tier | G1 x G2 x G3 | OS: HR=2.801 (95%CI:1.477-5.310), P=0.002 | NS |
| Zhou et al(23) | 2013 | 2004-2007 | LR | No previous treatment | 120 | NI | 3-tier | G1 x G2+G3 | OS: RR=0.840 (95%CI:0.543–1.298), P=0.432 | NI |
| Fu et al(24) | 2014 | 2008-2009 | LR | No previous treatment | 100 | NI | 3-tier | G1 x G2+G3 | OS was higher in more differentiated tumors (Cox regression analysis: HR=3.850, 95% CI=2.072-7.154, P<0.001) | NS |
| Lu et al(25) | 2013 | 2003-2006 | LR | No previous treatment | 101 | NI | 3-tier | G1 x G2+G3 | OS was higher in more differentiated tumors (Cox proportional hazard analysis: HR=0.578, 95% CI= 0.370-0.902, P=0.016) | OS: RR=1.194 (95%CI:0.725-1.965), P=0.486 |
| Zhou et al(26) | 2013 | 2004-2007 | LR | No previous treatment | 86 | NI | 3-tier | G1 x G2+G3 | OS: HR=0.712 (95%CI:0.427–1.188), P=0.118 | Not included |
| Dong et al(27) | 2012 | 2005-2009 | LR | No previous chemotherapy or radiotherapy | 120 | NI | 3-tier | G1 x G2 x G3 | — | NS |
| Huang et al(28) | 2011 | NI | LR | No previous chemotherapy | 87 | NI | 3-tier | NI | - | OS: RR=0.8102417 (95%CI:0.5072135-1.2943104), P=0.3785954 |
| Lim et al(29) | 2014 | November 1994-December 2010 | LR | NI | 616 | NI | 3-tier | G1+G2 x G3 | OS: P=0.3658 RFS: P=0.9419 | Not included |
| Zhang et al(30) | 2014 | April 2004-May 2007 | LR | NI | 90 | NI | 3-tier | G1 x G2 x G3 | Survival: 100% vs 62.2% vs 32.6%, P<0.001 | survival: HR=2.011 (95%CI: 1.026-3.943), P=0.042 |
| Liebl et al(31) | 2014 | January 1987-June 2009 | LR | no neoadjuvant therapy | 47 | NI | 4-tier | G1+G2 x G3+G4 | NI | Survival: HR=2.60 (95%CI: 1.03–6.52), P=0.042 |
| Cha et al(32) | 2015 | June 2004-December 2007 | LR | no prior treatment | 88 | NI | 4-tier | G1+G2 x G3+G4 | OS: HR=2.004 (95%CI: 1.086–3.697), P=0.026 DFS: HR=1.275 (95%CI: 0.743–2.188), P=0.377 | OS: NS DFS: not included |
| Xiong et al(33) | 2015 | 2005-2008 | LR | no prior chemotherapy | 98 | NI | 4-tier | G1+G2 x G3+G4 | OS: χ²=1.166, P=0.280 | OS: RR=0.850 (95%CI: 0.394–1.831), P=0.678 |
| Jiao et al(34) | 2015 | January 2004-December 2010 | LR | no prior treatment | 727 | NI | 4-tier | G1+G2 x G3+G4 | OS: HR=1.708 (95%CI: 1.366–2.135), P=0.0000026 DFS: HR=1.379 (95%CI: 1.014–1.875), P=0.041 | OS: HR=1.588 (95%CI: 1.256–2.008), P=0.00011 DFS: HR=1.432 (95%CI: 1.034–1.983), P=0.031 |
| Xie et al(35) | 2015 | 2006-2009 | LR | NI | 85 | NI | 4-tier | G1+G2 x G3+G4 | OS: P<0.001 Cumulative recurrence: P<0.001 | OS: HR=0.349 (95%CI: 0.151-0.803), P=0.013 Cumulative recurrence: HR=0.332 (95%CI: 0.144-0.762), P=0.009 |
| Wang et al(36) | 2015 | NI | LR | no prior treatment | 90 | NI | 4-tier | G1+G2 x G3+G4 | Survival: 71.4% vs 29.3%, χ²=15.902, P< 0.051? | Survival: HR=0.343 (95%CI: 0.599-0.817), P=0.023 |
| Yu et al(37) | 2011 | 1983-2005 | LR | NI | 1373 | NI | 4-tier | G1+G2 x G3+G4 | OS (5-year): 51.7% vs 46.1%, P=0.007 DFS (5-year): 33.3% vs 31%, P=0.012 | OS: HR 95%CI: 0.78–1.16, P=0.618 DFS: HR 95%CI: 0.80–1.15, P=0.670 |
| Pan et al(38) | 2014 | January 2008-December 2011 | LR | none | 90 | ES | 4-tier | G1+G2 x G3+G4 | OS: median survival (months) 35.3 vs 25.6, P<0.001 DFS: median survival (months) 29.5 vs 20, P<0.001 |  |
| Hu et al(39) | 2014 | 2005-2006 | LR | no prior treatment | 133 | ES | 4-tier | G1+G2 x G3+G4 | OS: HR=1.60 (95%CI: 0.97–2.64), P=0.066 TTR: HR=1.66 (95%CI: 1.01–2.74), P=0.048 | OS: not included TTR: HR=1.63 (95%CI: 0.96–2.79), P=0.073 |
|  |  | July 2010-June 2011 |  |  | 123 |  |  |  | OS: HR=1.71 (95%CI: 0.78–3.76), P=0.185 TTR: HR=1.51 (95%CI: 0.89–2.57), P=0.130 | not included |
| Huang et al(40) | 2014 | January 2006-December 2008 | LR | no prior chemotherapy or embolization | 86 | ES | 4-tier | G1+G2 x G3+G4 | NI | OS: HR=1.6 (95%CI: 0.7–3.7), P=0.256 DFS: HR=3.0 (95%CI: 1.7–5.3), P<0.001 |
| Li et al(41) | 2014 | 2007-2010 | LR | no prior treatment | 227 | ES | 4-tier | G1+G2 x G3+G4 | OS: P=0.154 RFS: P=0.354 | not included |
| Ruan et al(42) | 2014 | 2000-2003 | LR | no chemotherapy or radiotherapy | 82 | ES | 4-tier | G1+G2 x G3+G4 | OS: 70.3% vs 60.4% (2-year); 43.1% vs 36.8% (5-year), P=0.017 | OS: HR=0.739 (95%CI: 0.361,1.536), P=0.563 |
| Lin et al(43) | 2014 | 2000-2008 | LR | no prior treatment | 160 | ES | 4-tier | G1+G2 x G3+G4 | OS: P=0.001 | OS: RR=16.02, P=0.01 |
| Zhou et al(44) | 2015 | 2007-2008 | LR | NI | 452 | ES | 4-tier | G1+G2 x G3+G4 | TTR: HR=1.613 (95%CI: 1.248–2.084), P<0.001 OS: HR=1.792 (95%CI: 1.367–2.348), P<0.001 | TTR: HR=1.339 (95%CI: 1.029–1.742), P=0.030 OS: HR=1.424 (95%CI: 1.082–1.874), P=0.012 |
| Huang et al(45) | 2014 | 2009-2010 | LR | no chemotherapy or radiotherapy | 52 | ES | 4-tier | G1+G2 x G3+G4 | NI | OS: HR=3.038 (95%CI: 0.848-10.888), P=0.088 |
| Ji et al(46) | 2015 | June 2007-October 2009 | LR | NI | 84 | ES | 4-tier | G1+G2 x G3+G4 | DFS: 1-year: 54.8% vs 31.8% / 3-year: 41.9% vs 22.7% / 5-year: 35.1% vs 22.7%, P=0.16 OS: 1-year: 85.5% vs 68.2% / 3-year: 56.5% vs 36.4% / 5-year: 48.4% vs 27.3%, P=0.04 | DFS: not included OS: HR=0.498 (95%CI: 0.266-0.932), P=0.029 |
| Kuang et al(47) | 2015 | 2010-2012 | LR | no chemotherapy or radiotherapy | 107 | ES | 4-tier | G1+G2 x G3+G4 | OS: HR=3.740 (95%CI: 2.234, 6.264), P<0.001 | OS: HR=1.909 (95%CI: 1.030, 3.536), P=0.040 |
| Li et al(48) | 2015 | 2007-2010 | LR | no prior treatment | 259 | ES | 4-tier | G1+G2 x G3+G4 | OS: P=0.153 RFS: P=0.377 | OS: not included RFS: not included |
| Huang et al(49) | 2015 | July 1988-September 1996 | LR | no prior treatment | 227 | ES | 4-tier | G1+G2 x G3+G4 | DFS: HR=1.1894 (95%CI: 0.8347–1.6948), P=0.3371 OS: HR=1.0365 (95%CI: 0.7119–1.5092), P=0.8516 |  |
| Kim et al(50) | 2015 | March 1992-July 2004 | LR | NI | 136 | ES | 4-tier | G1+G2 x G3+G4 | Recurrence: HR=1.273 (95%CI: 0.974-1.665), P=0.0764 | Recurrence: HR=1.769 (95%CI: 1.159-2.701), P=0.00821 |
| Wang et al(51) | 2015 | 2006-2011 | LR | no prior treatment | 70 | ES | 4-tier | G1+G2 x G3+G4 | OS: HR=1.386 (95%CI: 1.042–1.844), P=0.025 DFS: HR=1.395 (95%CI: 1.051–1.852), P=0.021 | NS |
| Tang et al(52) | 2015 | March 2002-January 2007 | LR | no prior chemotherapy or embolization | 59 | ES | 4-tier | G1+G2 x G3+G4 | NI | Death: HR=5.075 (95%CI: 2.052-12.551), P<0.001 RFS: HR=5.001 (95%CI: 2.013-12.424), P=0.001 |
| Chen et al(53) | 2015 | April 2003-August 2010 | LR | no chemotherapy or radiotherapy | 118 | ES | 4-tier | G1 x G2 x G3+G4 | Survival: Χ²=46.112, P<0.001 | Survival: HR=1.6004 (95%CI: 1.0165–2.5197), P=0.0423 |
| Chen et al(54) | 2014 | April 2003-May 2009 | LR | No previous treatment | 103 | ES | 4-tier | G1 x G2 x G3+G4 | OS: χ²=104.273, P<0.001 | OS: HR=7.4760, (95%CI:3.7968-14.7206), P<0.0001 |
| Murakami et al(55) | 2014 | 1999-2010 | LR | No previous chemotherapy or radiotherapy | 181 | ES | 4-tier | G1+G2 x G3+G4 | — | NS |
| Lv et al(56) | 2013 | January 2008-December 2010 | LR | No previous treatment | 56 | ES | 4-tier | G1+G2 x G3+G4 | OS: median survival: low grade-39.7 months vs high grade-21.6, P=0.003 RFS: median survival: low grade-31 months vs high grade-18.2, P=0.013 | OS: HR=2.785, (95%CI:1.382-5.612), P=0.004 RFS: HR=2.138, (95%CI:1.139-4.014), P=0.018 |
| Zhou et al(57) | 2011 | 1995-2002 | LR | NI | 85 | ES | 4-tier | G1+G2 x G3+G4 | OS: 5-year survival: 78.4% vs 5.9%, P<0.001 RFS: 26.7% vs 0%, P<0.001 | OS: RR=5.968 (95%CI:2.375-14.998), P<0.001 RFS: RR=4.443, (95%CI:2.376-8.311), P<0.001 |
| Lee et al(58) | 2013 | 2001-2011 | LR | No previous treatment | 190 | ES | 4-tier | G1+G2 x G3+G4 | OS: 104.3±9.5 months vs 73.9±5, P=0.008 TTR: 73.1±11.1 vs 47.7±4.6, P=0.048 | OS: HR=3.42(95%CI:1.55-7.57), P=0.002 TTR: HR=2.02 (95%CI:1.16-3.52), P=0.013 |
| Tu et al(59) | 2014 | 2006-2008 | LR | NI | 60 | ES | 4-tier | G1+G2 x G3+G4 | — | OS: HR=0.771 (95%CI:0.322-1.846), P=0.560 DFS: HR=0.702 (95%CI:0.338-1.458), P=0.342 |
| Chen et al(60) | 2013 | April 2004-June 2008 | LR | No previous treatment | 101 | ES | 4-tier | G1 x G2 x G3+G4 | OS: P<0.001 | OS: HR=3.7803 (95%CI:2.1642–6.6031), P<0.0001 |
| Zhao et al(61) | 2012 | March 2001-March 2006 | LR | No previous treatment | 168 | ES | 4-tier | G1+G2 x G3+G4 | OS: P=0.819 RFS: P=0.060 | NI |
| Zhang et al(62) | 2012 | 2000-2005 | LR | NI | 104 | ES | 4-tier | G1+G2 x G3+G4 | OS: RR=1.72 (95%CI:1.03–2.87), P=0.037 | NS |
| Hu et al(63) | 2014 | 02/2006–10/2007 and 02/2013–04/2013 | LR | No previous chemotherapy | 128 | ES | 4-tier | G1+G2 x G3+G4 | OS: HR=2.281 (95%CI:1.495-3.480), P<0.001 DFS: HR=2.061 (95%CI:1.380-3.078), P<0.001 | OS: HR=1.77 (95%CI:1.113-2.813), P=0.016 |
| Wang et al(64) | 2014 | NI | LR | NI | 67 | ES | 4-tier | G1+G2 x G3+G4 | OS: G1-44.9% vs G2-40%, P=0.723 | OS: HR=1.8 (95%CI:1.07-3.03), P=0.026 |
| Liu et al(65) | 2013 | 2002-2005 | LR | No previous treatment | 134 | ES | 4-tier | G1+G2 x G3+G4 | OS: HR=1.60 (95%CI:1.05-2.43), P=0.03 TTR: HR=1.71, (95%CI:1.10-2.65), P=0.018 | OS: HR=1.92 (95%CI:1.25–2.30), P=0.003 TTR: HR=2.03, (95%CI:1.29-3.18), P=0.002 |
| Lv et al(66) | 2013 | 2006-2010 | LR | No previous treatment | 71 | ES | 4-tier | G1+G2 x G3+G4 | OS: mean survival(months): 41 (95%CI:32.950–50.085) vs 20 (15.715–23.624) P<0.001 RFS: 38 (29.212–47.114) vs 17 (13.330–21.133), p<0.001 | OS: HR=3.202 (95%CI:1.718-5.966), P<0.001 DFS HR=2.823 (95%CI:1.576-5.056), p<0.001 |
| Guo et al(67) | 2012 | 2001-2006 | LR | No previous chemotherapy or radiotherapy | 156 | ES | 4-tier | G1+G2 x G3+G4 | OS: RR=5.465 (95%CI:2.609-9.088), P=0.006 DFS: RR=5.951 (95%CI:2.605-9.466), P=0.005 | OS: 1.653 (95%CI:0.609–4.088), P=0.068 DFS: HR=2.551 (95%CI:1.207-6.466), P=0.031 |
| Chen et al(68) | 2012 | 1982-1998 | LR | No previous chemotherapy or chemoembolization | 172 | ES | 4-tier | G1+G2 x G3+G4 | — | NS |
| Chen et al(69) | 2012 | NI | LR | NI | 120 | ES | 4-tier | G1+G2 x G3+G4 | — | OS: RR=13.64 (95%CI:2.55-72.99), P=0.002 DFS: RR=3.32 (95%CI:0.54–19.04), P=0.203 |
| Park and Yu(70) | 2013 | 1997-2004 | LR | No previous treatment | 370 | ES | 4-tier | G1+G2 x G3+G4 | OS: HR=1.486 (95%CI:1.044-2.115), P=0.028) RFS: HR=1.177 (95%CI:0.880–1.575), P=0.273 | NS |
| Noda et al(71) | 2012 | NI | LR | NI | 139 | ES | 4-tier | G1+G2 x G3+G4 | DFS: G1+G2-55.5 months vs G3+G4-35.4 months, P=0.049 | DFS: HR=1.292 (95%CI:0.849-1.964), P=0.232 |
| Zhang et al(72) | 2014 | December 2009-June 2010 | LR | No previous chemotherapy or chemoembolization | 58 | ES | 4-tier | G1+G2 x G3+G4 | OS was higher in more differentiated tumors (log-rank test: χ2‑value=12.014 P=0.001) | Grading was an independent predictor of OS (Cox proportional hazard regression model: HR=2.393, 95%CI=1.676‑8.385, P=0.001) |
| Shindoh et al(73) | 2013 | 1981-2011 | LR | NI | 927 | ES | 4-tier | G1+G2 x G3+G4 | OS was higher in more differentiated tumors (HR=1.5, 95%CI=1.22–1.84, P=0.0001) | Grading was an independent predictor of OS (HR 1.29, 95%CI 1.01-1.64, P = 0.04) |
|  |  |  |  |  | 148 |  |  |  | OS: HR=1.27 (95%CI:0.6–2.47), P=0.51 | — |
| Huang et al(74) | 2012 | 2004-2007 | LR | No previous treatment | 112 | ES | 4-tier | G1 x G2 x G3 | OS: HR=1.419 (95%CI:1.005-2.003), P=0.046 | NS |
| Yuan et al(75) | 2013 | 1982-1998 | LR | No previous treatment | 259 | ES | 4-tier | G1 x G2+G3+G4 | ETR: OR=1.70 (95%CI:1.01-2.89), P=0.0456 | ETR: HR (low grade/high grade) =0.7895 (95%CI:0.5106–1.2223), P=0.2898 |
| Zhou et al(76) | 2013 | 1995-2002 | LR | NI | 156 | ES | 4-tier | G1+G2 x G3+G4 | OS: mean±SE: 80±5 vs 21±2 months, P<0.001 DFS: 57±5 vs 13±1, P<0.001 | OS: RR=5.944 (95%CI:3.167-11.156), P<0.001 DFS: RR=6.175 (95%CI=3.78-10.087), P<0.001 |
| Chen et al(77) | 2013 | 2000-2008 | LR | NI | 303 | ES | 4-tier | G1+G2 x G3+G4 | OS: HR=2.16, (95%CI:1.39-3.34), P=0.0001 | OS: HR=1.62 (95%CI:1.04–2.55), P=0.0342 |
| Han et al(78) | 2013 | January 1996-March 2010 | LR | NI | 625 | ES | Homogeneous: HG1, HG2, HG3, HG4 Mixed: M1(worst grade G2), M2(worst grade G3), M3 (worst grade G4) | OTHER | OS: HG2 vs M2: HG2 vs M2: 1-year:95.4% vs 93.6%, 3-year:83.2% vs 75.4%, 5-year:71.4% vs 55.1%, P=0.025 / G1+G2 vs G3+G4 (worst grade): 1-year:95.1% vs 86.8%, 3-year:83.9% vs 68.6%, 5-year:74.1% vs 54.6%, P<0.001 DFS: HG2 vs M2: 1-year:79.5% vs 66.3%, 3-year:62.4% vs 47.5%, 5-year:52.2% vs 43.5%, P=0.004 / G1+G2 vs G3+G4 (worst grade): 1-year:81.4% vs 62.2%, 3-year:61.7% vs 44.3%, 5-year:52.1% vs 40.3%, P<0.001 | G3+G4/G1+G2: OS: RR-95%CI:1.307-2.395, P<0.001 DFS: RR–95%CI:1.305-1.805, P=0.028 HG2 vs M2: OS: RR-95%CI:1.096-2.451, P=0.016 DFS:RR-95%CI:1.081-2.209, P=0.017 |
| Xu et al(79) | 2012 | August 2002-December 2009 | LR | No palliative (sorafenib, TACE) treatments | 41 | ES | 4-tier | G1+G2 x G3+G4 | Survival: P=0.28 | Not included |
|  |  |  |  |  | 73 |  |  |  | Survival: P<0.01 | Survival: P<0.01 |
| Qiu et al(80) | 2014 | January 2003-December 2004 | LR | No previous treatment | 103 | ES | 4-tier | G1+G2 x G3+G4 | OS: HR=1.421 (95%CI:1.109-1.821), P=0.005 | OS: HR=1.365 (95%CI:1.062-1.753), P=0.015 |
| Hu et al(81) | 2014 | 2005-2008 | LR | No previous chemotherapy | 103 | ES | 4-tier | G1+G2 x G3+G4 | Survival: 51.8% vs 40.4%, χ²=1.325, P=0.250 | OS: HR=0.649 (95%CI: 0.323–1.302), P=0.223 |
| Zhu et al(82) | 2014 | 2006-2010 | LR | no chemotherapy or radiotherapy | 72 | ES | 4-tier | G1+G2 x G3+G4 | OS: low grade 32.950-50.085 months(95%CI) vs high grade 16.016-23.947 months (95%CI), P<0.001 DFS: low grade 29.212-47.114 months(95%CI) vs high grade 14.001-22.435 months (95%CI), P<0.001 | OS: HR=1.772 (95%CI:1.301-2.415), P<0.001 DFS: HR=1.625 (95%CI:1.215-2.173), P=0.001 |
| Kim et al(83) | 2014 | January 2005-December 201 | LR | No previous locoregional therapy | 360 | ES | 4-tier | G1+G2 x G3+G4 | Recurrence: OR=1.242 (95%CI:0.729–2.116), P=0.425 | Not included |
|  |  |  |  |  | 103 |  |  |  | Recurrence: OR=1.539 (95%CI:0.544–4.356), P=0.416 | Not included |
| Liu et al(84) | 2011 | January 2003-December 2008 | LR | No previous treatment | 151 | ES | 4-tier | G1+G2 x G3+G4 | OS: mean survival(95%CI): 50 (43–57) vs 41 (36–46) P=0.137 DFS: 32 (26–38) vs 27 (23–31), p=0.234 | Not included |
| Shin et al(85) | 2011 | 1995-2004 | LR | NI | 412 | ES | 4-tier | NI | DFS: P=0.180 | NI |
| Yuan et al(86) | 2014 | January 1985-August 2008 | LR | No previous treatment | 159 | ES | 4-tier | G1 x G2+G3+G4 | NI/significant (was included in multivariate analysis) | OS: HR=1.4997 (95%CI:0.8704–2.5841), P=0.1443 |
| Zhou et al(87) | 2014 | NI | LR | NI | 152 | ES | 4-tier | G1+G2 x G3+G4 | TSS: mean±SE (95%CI): low grade - 81±5(71–91) vs high grade – 18±2(14–22), P<0.001 TFS: low grade - 50±7(35–65) vs high grade – 12±1(10–14), P<0.001 Recurrence: low grade-49.4% vs high grade-89.2%, P<0.001 Early recurrence: low grade-11.5% vs high grade-52.3%, P<0.001 | TSS: HR=4.980 (95%CI:2.667–9.300), P<0.001 TFS: HR=5.440 (95%CI:3.337–8.867), P<0.001 Recurrence: HR=6.750 (95%CI:2.649–17.201), P<0.001 Early recurrence: HR=6.527 (95%CI:2.769–15.383), P<0.001 |
| Guo et al(88) | 2014 | March 2001-February 2006 | LR | no chemotherapy or radiotherapy | 130 | ES | 3-tier | G1 x G2 x G3 | - | OS: HR=1.563 (95%CI: 0.609-4.088), P=0.081 DFS: HR=1.551 (95%CI: 0.607–4.466), P=0.086 |
| Chang et al(89) | 2015 | 2005-2006 | LR | NI | 86 | ES | 3-tier | G1 x G2 x G3 | Survival: χ2 value=14.027, OR=0.312 (95%CI: 0.079–0.518), P=0.001 | Survival: HR=0.451 (95%CI: 0.254–0.801), P=0.007 |
| Zhang et al(90) | 2016 | November 2007-December 2008 | LR | no prior treatment | 149 | ES | 3-tier | G1 x G2 x G3 | OS: HR=1.437 (95%CI: 1.011–2.041), P=0.043 | OS: HR=1.906 (95%CI: 1.333–2.727), P<0.001 |
| Yang et al(91) | 2015 | 2008-2013 | LR | no prior chemotherapy | 58 | ES | 3-tier | G1 x G2 x G3 | Survival: HR=0.58 (95%CI: 0.320–1.054), P=0.07 | - |
| Ha et al(92) | 2015 | July 2000-May 2006 | LR | no prior chemotherapy | 226 | ES | 3-tier | G1+G2 x G3 | early recurrence: OR= 1.929 (95%CI: 0.995-3.739), P=0.052 RFS: HR=1.618 (95%CI: 1.137-2.302), P=0.007 OS: HR=1.589 (95%CI: 1.038-2.434), P=0.033 | Early recurrence: not included RFS: HR=0.933 (95%CI: 0.626-1.390), P=0.732 OS: HR=0.853 (95%CI: 0.530-1.371), P=0.510 |
| Ha et al(93) | 2015 | July 2000-May 2006 | LR | no chemotherapy, radiotherapy or ablation | 282 | ES | 3-tier | G1+G2 x G3 | NI | DFS: HR=1.403 (95%CI: 0.830-2.372), P=0.206 DSS: HR=1.336 (95%CI: 0.712-2.509), P=0.367 |
| Cong et al(94) | 2014 | 2004-2005 | LR | No previous chemotherapy, radiation or immunotherapy | 96 | ES | 3-tier | G1 x G2 x G3 | OS: G1-82.6% vs G2-27.6% vs G3-6.7%, P<0.001 | OS: HR=2.476, (95%CI:1.665-3.683), P<0.001 |
| Pedica et al(95) | 2013 | January 2006-December 2009 | LR | NI | 65 | ES | 3-tier | G1 x G2 x G3 | OS: G1-33.89 months (95%CI:24.77–43.01) vs G2-37.35 months (95%CI:32.02–42.68) vs G3-27.17 (95%CI:20.41–33.94), P=0.660 DFS: G1-15.61 months (95%CI:6.28–24.94) vs G2-23.51 months (95%CI:18.41–28.60) vs G3-22.37 (95%CI:14.98–29.74), P=0.438 | Not included |
| Guo et al(96) | 2012 | 2001-2006 | LR | No previous treatment | 130 | ES | 3-tier | G1 x G2 x G3 | — | OS: HR=1.563 (95%CI:0.609-4.088), P=0.081 DFS: HR=1.551 (95%CI:0.607-4.466), P=0.086 |
| Chen et al(97) | 2013 | March 2005-2007 | LR | No previous chemotherapy or radiotherapy | 134 | ES | 3-tier | G1 x G2 x G3 | — | OS: HR=1.79 (95%CI=1.49-2.34), P=0.011 |
| Mizuno et al(98) | 2012 | 2000-2009 | LR | NI | 74 | ES | 3-tier | G1 x G2 x G3 | OS: HR=1.33 (95%CI:0.60–2.96), P=0.485 | NI |
| Pan et al(99) | 2013 | 2002-2007 | LR | No previous chemotherapy or radiotherapy | 180 | ES | 3-tier | G1 x G2 x G3 | OS: HR=1.96 (95%CI:2.48-6.94), P=0.004 DFS: HR=1.83 (95%CI:2.16-6.15), P=0.016 | NS |
| Wu et al(100) | 2014 | September 2003-September 2010 | LR | no chemotherapy, radiotherapy or previous surgery | 156 | WHO | 3-tier | G1 x G2+G3 | OS: HR=1.442 (95%CI=0.744-2.797), P=0.279 | OS: HR=1.757 (95%CI: 0.843–3.664), P=0.133 |
| Sasaki et al(101) | 2015 | 1994-2012 | LR | NI | 233 | WHO | 3-tier | G1+G2 x G3 | Overall recurrence: 53.3% vs 76% (P=0.01) Recurrence within 1 year: 11.1% vs 24% (P=0.06) Recurrence within 2 years: 24.6% vs 38% (P=0.14) Advanced recurrence within 1 year: 2% vs 24% (P<0.01) Advanced recurrence within 2 years: 3.5% vs 24% (P<0.01) | Recurrence: HR=1.99 (95%CI: 1.25–3.16), P<0.01 |
| Sasaki et al(102) | 2014 | January 1995-December 2010 | LR | 13.11% (TACE) | 427 | WHO | NP (containing only G1 and G2), PC (containing G3, predominant G1 or G2), PD (predominant G3) | OTHER | OS: 5-year: NP-82%, PC-55%, PD-77%; 10-year: 41%, 63%, 50% (NP vs PC: P<0.01 / NP vs PD: P<0.01 / PC vs PD: P=0.99) RFS: 1-year: NP-85%, PC-62%, PD-47%; 3-year: 77%, 40%, 31%; 5-year: 68%, 40%, 33% (NP vs PC: P=0.01 / NP vs PD: P<0.01 / PC vs PD: P=0.51) | OS: HR (presence of poorly differentiated component) =1.56 (95%CI:1.03-2.19), P=0.02 RFS: HR=1.66 (95%CI:1.25-2.21), P<0.01 |
| Lu et al(103) | 2012 | 2002-2003 | LR | NI | 60 | WHO | 3-tier | G1+G2 x G3 | OS: HR=1.140 (95%CI:0.654–1.989), P=0.634 | NI |
| Geng et al(104) | 2012 | January 2006 – September 2010 | LR | NI | 85 | WHO | 3-tier | G1 x G2 x G3 | OS: HR=1.388 (95%CI:0.777-2.482), P=0.268 | OS: HR=1.462 (95%CI:0.715-2.993), P=0.298 |
| Chao et al(105) | 2014 | 2001-2004 | LR | no chemotherapy or radiotherapy | 129 | WHO | 3-tier | OTHER | OS: HR=1.264 (95%CI: 0.697–2.291), P=0.440 | Not included |
|  |  |  |  |  |  |  |  | OTHER | OS: HR=1.759 (95%CI: 0.845–3.661), P=0.131 | Not included |
| Liu et al(106) | 2011 | January 2001-December 2002 | LR | No previous treatment | 400 | WHO | 3-tier | OTHER | OS: G1x G2-HR=1.35 (95%CI:0.53–9.17), P=0.896 G1xG3-HR=1.32 (95%CI:0.74–7.59), P=0.514 Early recurrence: G1x G2-HR=1.32 (95%CI:0.64–7.35), P=0.538 G1xG3-HR=1.36 (95%CI:1.87–15.9), P=0.571 | Not included |
| Lu et al(107) | 2011 | NI | LR | NI | 55 | WHO | 3-tier | G1+G2 x G3 | OS: HR=1.094 (95%CI:0.625–1.915), P=0.754 | Not included |
| Luo et al(108) | 2012 | 1999-2002 | LR | No previous treatment | 99 | WHO | 4-tier | G1+G2 x G3+G4 | OS: HR=0.551 (95% CI: 0.335–0.906), P=0.019 | OS: HR=0.576 (95%CI:0.346–0.961), P=0.035 |
| Jia et al(109) | 2014 | January 1999-December 2004 | LR | No previous treatment | 213 | WHO | 4-tier | G1+G2 x G3+G4 | PFS: HR=1.335 (95%CI:0.966-1.847), P=0.080 | NI |
| Harimoto et al(110) | 2015 | January 2004-December 2012 | LR | Combined ablation therapy | 966 | OTHERS | 3-tier | G1+G2 x G3 | DFS: HR=1.883 (95%CI: 1.447-2.392), P<0.001 RFS: NS, HR=1.109 (95%CI: 0.914-1.347), P=0.293 | DFS: HR=1.490 (95%CI: 1.126-1.972), P=0.005 RFS: not included |
| Zhang et al(111) | 2014 | 2006-2008 | LR | none | 167 | OTHERS | 3-tier | G1 x G2 x G3 | - | OS: HR=1.443 (95%CI: 0.969–2.184), P=0.089 RFS: HR=1.454 (95%CI: 0.977–2.171), P=0.074 |
| Huang et al(112) | 2013 | January 2006-December 2010 | LR | NI | 70 | OTHERS | 3-tier | G1 x G2+G3 | OS: RR=2.828 (95%CI:1.380-5.795), P=0.005 | NS |
| Cao et al(113) | 2012 | January 2003-December 2006 | LR | NI | 80 | OTHERS | 3-tier | G1 x G2 x G3 | OS: 77.8% vs 60% vs 39.1%, P=0.023 | OS: HR=2.4567 (95%CI:1.3546-4.8765), P=0.002 |
| Wang et al(114) | 2011 | 2004-2006 | LR | NI | 36 | OTHERS | 3-tier | G1 x G2 x G3 | OS: HR=3.521 (95%CI: 0.466-26.61), P=0.223 Recurrence: HR=0.938 (95%CI:0.462-1.904), P=0.860 | — |

Abbreviations: CI: confidence interval; CS: cumulative survival; DFS: disease-free survival; ES Edmondson & Steiner; ETR: early tumor recurrence; HR: hazard ratio; LR: liver resection; LT: liver transplantation; NI: not informed; NP: noon-poor; NS: non-significant; OR: overall survival; PC: poorly-containing; PD: poorly-differentiated; PEI: percutaneous ethanol injection; PFS: progression-free survival; RFA: radiofrequency ablation; RFS: recurrence-free survival; RR: relative risk; TACE: transcatheter arterial chemoembolization; TFS: tumor-free survival; TSS: tumor-specific survival; TTR: time to recurrence; WHO: World Health Organization.

**REFERENCE:**

1. Shamsaeefar A, Nikeghbalian S, Kazemi K, Gholami S, Motazedian N, Motazedian N, Fallahzadeh ME, Moini M, Gramizadeh B, Malekhosseini SA. Predictors of tumor-free survival after liver transplant in patient with hepatocellular carcinoma. *Exp Clin Transplant Off J Middle East Soc Organ Transplant* (2015) **13 Suppl 1**:139–144.

2. Andreou A, Gül S, Pascher A, Schöning W, Al-Abadi H, Bahra M, Klein F, Denecke T, Strücker B, Puhl G, et al. Patient and tumour biology predict survival beyond the Milan criteria in liver transplantation for hepatocellular carcinoma. *HPB* (2015) **17**:168–175. doi:10.1111/hpb.12345

3. Park M-S, Lee K-W, Yi N-J, Choi YR, Kim H, Hong G, Suh K-S, Kwon C-HD, Joh J-W, Lee S-K. Optimal tailored screening protocol after living donor liver transplantation for hepatocellular carcinoma. *J Korean Med Sci* (2014) **29**:1360–1366. doi:10.3346/jkms.2014.29.10.1360

4. Iguchi T, Shirabe K, Aishima S, Wang H, Fujita N, Ninomiya M, Yamashita Y, Ikegami T, Uchiyama H, Yoshizumi T, et al. New Pathologic Stratification of Microvascular Invasion in Hepatocellular Carcinoma: Predicting Prognosis After Living-donor Liver Transplantation. *Transplantation* (2015) **99**:1236–1242. doi:10.1097/TP.0000000000000489

5. Hoyos S, Escobar J, Cardona D, Guzmán C, Mena Á, Osorio G, Pérez C, Restrepo JC, Correa G. Factors associated with recurrence and survival in liver transplant patients with HCC--a single center retrospective study. *Ann Hepatol* (2015) **14**:58–63.

6. Wang L-Y, Zheng S-S, Xu X, Wang W-L, Wu J, Zhang M, Shen Y, Yan S, Xie H-Y, Chen X-H, et al. A score model for predicting post-liver transplantation survival in HBV cirrhosis-related hepatocellular carcinoma recipients: a single center 5-year experience. *Hepatobiliary Pancreat Dis Int HBPD INT* (2015) **14**:43–49.

7. Varona MA, Soriano A, Aguirre-Jaime A, Garrido S, Oton E, Diaz D, Portero J, Bravo P, Barrera MA, Perera A. Risk factors of hepatocellular carcinoma recurrence after liver transplantation: accuracy of the alpha-fetoprotein model in a single-center experience. *Transplant Proc* (2015) **47**:84–89. doi:10.1016/j.transproceed.2014.12.013

8. Cui X, Li Z, Gao P-J, Gao J, Zhu J-Y. Prognostic value of glypican-3 in patients with HBV-associated hepatocellular carcinoma after liver transplantation. *Hepatobiliary Pancreat Dis Int HBPD INT* (2015) **14**:157–163.

9. Wang P, Song W, Li H, Wang C, Shi B, Guo W, Zhong L. Association between donor and recipient smoothened gene polymorphisms and the risk of hepatocellular carcinoma recurrence following orthotopic liver transplantation in a Han Chinese population. *Tumour Biol J Int Soc Oncodevelopmental Biol Med* (2015) **36**:7807–7815. doi:10.1007/s13277-015-3370-x

10. An HJ, Jang JW, Bae SH, Choi JY, Yoon SK, Lee MA, You YK, Kim DG, Jung ES. Serum C-reactive protein is a useful biomarker for predicting outcomes after liver transplantation in patients with hepatocellular carcinoma. *Liver Transplant Off Publ Am Assoc Study Liver Dis Int Liver Transplant Soc* (2012) **18**:1406–1414. doi:10.1002/lt.23512

11. Wan P, Xia Q, Zhang J-J, Li Q-G, Xu N, Zhang M, Chen X-S, Han L-Z. Liver transplantation for hepatocellular carcinoma exceeding the Milan criteria: a single-center experience. *J Cancer Res Clin Oncol* (2014) **140**:341–348. doi:10.1007/s00432-013-1576-0

12. Hua L, Wang C-Y, Yao K-H, Chen J-T, Zhang J-J, Ma W-L. High expression of long non-coding RNA ANRIL is associated with poor prognosis in hepatocellular carcinoma. *Int J Clin Exp Pathol* (2015) **8**:3076–3082.

13. Zhu G, Shi W, Fan H, Zhang X, Xu J, Chen Y, Xu Z, Tao T, Cheng C. HES5 promotes cell proliferation and invasion through activation of STAT3 and predicts poor survival in hepatocellular carcinoma. *Exp Mol Pathol* (2015) **99**:474–484. doi:10.1016/j.yexmp.2015.09.002

14. Gu X, Fu M, Ge Z, Zhan F, Ding Y, Ni H, Zhang W, Zhu Y, Tang X, Xiong L, et al. High expression of MAGE-A9 correlates with unfavorable survival in hepatocellular carcinoma. *Sci Rep* (2014) **4**:6625. doi:10.1038/srep06625

15. Kishida N, Hibi T, Itano O, Okabayashi K, Shinoda M, Kitago M, Abe Y, Yagi H, Kitagawa Y. Validation of hepatectomy for elderly patients with hepatocellular carcinoma. *Ann Surg Oncol* (2015) **22**:3094–3101. doi:10.1245/s10434-014-4350-x

16. Chen R, Zhou X, Yu Z, Liu J, Huang G. Low Expression of LDHB Correlates With Unfavorable Survival in Hepatocellular Carcinoma: Strobe-Compliant Article. *Medicine (Baltimore)* (2015) **94**:e1583. doi:10.1097/MD.0000000000001583

17. An S-L, Xiao T, Wang L-M, Rong W-Q, Wu F, Feng L, Liu F-Q, Tian F, Wu J-X. Prognostic Significance of Preoperative Serum Alpha- fetoprotein in Hepatocellular Carcinoma and Correlation with Clinicopathological Factors: a Single-center Experience from China. *Asian Pac J Cancer Prev APJCP* (2015) **16**:4421–4427.

18. Lu P, Wang Z-P, Dang Z, Zheng Z-G, Li X, Zhou L, Ding R, Yue S-Q, Dou K-F. Expression of NEDD9 in hepatocellular carcinoma and its clinical significance. *Oncol Rep* (2015) **33**:2375–2383. doi:10.3892/or.2015.3863

19. Zhang S-H, Qian Y-M, Liu A-W, Cai J, Zhao X-L, Wei J-J, Zhu M-H. Clinicopathologic significance and function of S-phase kinase-associated protein 2 overexpression in hepatocellular carcinoma. *Hum Pathol* (2012) **43**:1084–1093. doi:10.1016/j.humpath.2011.08.019

20. Faber W, Stockmann M, Schirmer C, Möllerarnd A, Denecke T, Bahra M, Klein F, Schott E, Neuhaus P, Seehofer D. Significant impact of patient age on outcome after liver resection for HCC in cirrhosis. *Eur J Surg Oncol J Eur Soc Surg Oncol Br Assoc Surg Oncol* (2014) **40**:208–213. doi:10.1016/j.ejso.2013.10.018

21. Ni W, Chen B, Zhou G, Lu C, Xiao M, Guan C, Zhang Y, He S, Shen A, Ni R. Overexpressed nuclear BAG-1 in human hepatocellular carcinoma is associated with poor prognosis and resistance to doxorubicin. *J Cell Biochem* (2013) **114**:2120–2130. doi:10.1002/jcb.24560

22. Wei R-R, Zhang M-Y, Rao H-L, Pu H-Y, Zhang H-Z, Wang H-Y. Identification of ADH4 as a novel and potential prognostic marker in hepatocellular carcinoma. *Med Oncol Northwood Lond Engl* (2012) **29**:2737–2743. doi:10.1007/s12032-011-0126-3

23. Zhou L, Zhang N, Li Q-J, Sun W, Zhang Y, Wang D-S, Dou K-F. Associations between high levels of Notch1 expression and high invasion and poor overall survival in hepatocellular carcinoma. *Tumour Biol J Int Soc Oncodevelopmental Biol Med* (2013) **34**:543–553. doi:10.1007/s13277-012-0580-3

24. Fu R-D, Qiu C-H, Chen H-A, Zhang Z-G, Lu M-Q. Retinoic acid receptor-related receptor alpha (RORalpha) is a prognostic marker for hepatocellular carcinoma. *Tumour Biol J Int Soc Oncodevelopmental Biol Med* (2014) **35**:7603–7610. doi:10.1007/s13277-014-2007-9

25. Lu C-Y, Yang Z-X, Zhou L, Huang Z-Z, Zhang H-T, Li J, Tao K-S, Xie B-Z. High levels of EphA3 expression are associated with high invasive capacity and poor overall survival in hepatocellular carcinoma. *Oncol Rep* (2013) **30**:2179–2186. doi:10.3892/or.2013.2679

26. Zhou L, Zhang N, Song W, You N, Li Q, Sun W, Zhang Y, Wang D, Dou K. The significance of Notch1 compared with Notch3 in high metastasis and poor overall survival in hepatocellular carcinoma. *PloS One* (2013) **8**:e57382. doi:10.1371/journal.pone.0057382

27. Dong W-W, Mou Q, Chen J, Cui J-T, Li W-M, Xiao W-H. Differential expression of Rab27A/B correlates with clinical outcome in hepatocellular carcinoma. *World J Gastroenterol* (2012) **18**:1806–1813. doi:10.3748/wjg.v18.i15.1806

28. Huang X, Qian X, Cheng C, He S, Sun L, Ke Q, Zhang L, Pan X, He F, Wang Q, et al. Expression of Pirh2, a p27(Kip1) ubiquitin ligase, in hepatocellular carcinoma: correlation with p27(Kip1) and cell proliferation. *Hum Pathol* (2011) **42**:507–515. doi:10.1016/j.humpath.2010.04.021

29. Lim C, Mise Y, Sakamoto Y, Yamamoto S, Shindoh J, Ishizawa T, Aoki T, Hasegawa K, Sugawara Y, Makuuchi M, et al. Above 5 cm, size does not matter anymore in patients with hepatocellular carcinoma. *World J Surg* (2014) **38**:2910–2918. doi:10.1007/s00268-014-2704-y

30. Zhang J, Lu C, He S, Wan C, Zhang Y, Cheng C. Decreased expression of Small glutamine-rich tetratricopeptide repeat-containing protein (SGT) correlated with prognosis of Hepatocellular carcinoma. *Neoplasma* (2014) **61**:83–89.

31. Liebl F, Demir IE, Mayer K, Schuster T, DʼHaese JG, Becker K, Langer R, Bergmann F, Wang K, Rosenberg R, et al. The impact of neural invasion severity in gastrointestinal malignancies: a clinicopathological study. *Ann Surg* (2014) **260**:900-907; discussion 907-908. doi:10.1097/SLA.0000000000000968

32. Cha Y-L, Li P-D, Yuan L-J, Zhang M-Y, Zhang Y-J, Rao H-L, Zhang H-Z, Zheng XFS, Wang H-Y. EIF4EBP1 overexpression is associated with poor survival and disease progression in patients with hepatocellular carcinoma. *PloS One* (2015) **10**:e0117493. doi:10.1371/journal.pone.0117493

33. Xiong Y, Hu B, Wei L, Jiang D, Zhu M. Upregulated expression of polycomb protein Ring1 contributes to poor prognosis and accelerated proliferation in human hepatocellular carcinoma. *Tumour Biol J Int Soc Oncodevelopmental Biol Med* (2015) **36**:9579–9588. doi:10.1007/s13277-015-3721-7

34. Jiao H-K, Xu Y, Li J, Wang W, Mei Z, Long X-D, Chen G-Q. Prognostic significance of Cbx4 expression and its beneficial effect for transarterial chemoembolization in hepatocellular carcinoma. *Cell Death Dis* (2015) **6**:e1689. doi:10.1038/cddis.2015.57

35. Xie C, Lu Z, Liu G, Fang Y, Liu J, Huang Z, Wang F, Wu X, Lei X, Li X, et al. Numb downregulation suppresses cell growth and is associated with a poor prognosis of human hepatocellular carcinoma. *Int J Mol Med* (2015) **36**:653–660. doi:10.3892/ijmm.2015.2279

36. Wang G, Chen J-H, Qiang Y, Wang D-Z, Chen Z. Decreased STAT4 indicates poor prognosis and enhanced cell proliferation in hepatocellular carcinoma. *World J Gastroenterol* (2015) **21**:3983–3993. doi:10.3748/wjg.v21.i13.3983

37. Yu M-C, Chan K-M, Lee C-F, Lee Y-S, Eldeen FZ, Chou H-S, Lee W-C, Chen M-F. Alkaline phosphatase: does it have a role in predicting hepatocellular carcinoma recurrence? *J Gastrointest Surg Off J Soc Surg Aliment Tract* (2011) **15**:1440–1449. doi:10.1007/s11605-011-1537-3

38. Pan T-T, Jia W-D, Yao Q-Y, Sun Q-K, Ren W-H, Huang M, Ma J, Li J-S, Ma J-L, Yu J-H, et al. Overexpression of HOXA13 as a potential marker for diagnosis and poor prognosis of hepatocellular carcinoma. *Tohoku J Exp Med* (2014) **234**:209–219.

39. Hu B, Yang X-R, Xu Y, Sun Y-F, Sun C, Guo W, Zhang X, Wang W-M, Qiu S-J, Zhou J, et al. Systemic immune-inflammation index predicts prognosis of patients after curative resection for hepatocellular carcinoma. *Clin Cancer Res Off J Am Assoc Cancer Res* (2014) **20**:6212–6222. doi:10.1158/1078-0432.CCR-14-0442

40. Huang Y, Guo W, Kan H. TPX2 is a prognostic marker and contributes to growth and metastasis of human hepatocellular carcinoma. *Int J Mol Sci* (2014) **15**:18148–18161. doi:10.3390/ijms151018148

41. Li L, Yan J, Xu J, Liu C-Q, Zhen Z-J, Chen H-W, Ji Y, Wu Z-P, Hu J-Y, Zheng L, et al. CXCL17 expression predicts poor prognosis and correlates with adverse immune infiltration in hepatocellular carcinoma. *PloS One* (2014) **9**:e110064. doi:10.1371/journal.pone.0110064

42. Ruan J, Zheng H, Fu W, Zhao P, Su N, Luo R. Increased expression of cathepsin L: a novel independent prognostic marker of worse outcome in hepatocellular carcinoma patients. *PloS One* (2014) **9**:e112136. doi:10.1371/journal.pone.0112136

43. Lin Y, Lin G, Fang W, Zhu H, Chu K. Increased expression of annexin A1 predicts poor prognosis in human hepatocellular carcinoma and enhances cell malignant phenotype. *Med Oncol Northwood Lond Engl* (2014) **31**:327. doi:10.1007/s12032-014-0327-7

44. Zhou S-L, Zhou Z-J, Hu Z-Q, Li X, Huang X-W, Wang Z, Fan J, Dai Z, Zhou J. CXCR2/CXCL5 axis contributes to epithelial-mesenchymal transition of HCC cells through activating PI3K/Akt/GSK-3β/Snail signaling. *Cancer Lett* (2015) **358**:124–135. doi:10.1016/j.canlet.2014.11.044

45. Huang G-M, Jiang Q-H, Cai C, Qu M, Shen W. SCD1 negatively regulates autophagy-induced cell death in human hepatocellular carcinoma through inactivation of the AMPK signaling pathway. *Cancer Lett* (2015) **358**:180–190. doi:10.1016/j.canlet.2014.12.036

46. Ji F, Fu S-J, Shen S-L, Zhang L-J, Cao Q-H, Li S-Q, Peng B-G, Liang L-J, Hua Y-P. The prognostic value of combined TGF-β1 and ELF in hepatocellular carcinoma. *BMC Cancer* (2015) **15**:116. doi:10.1186/s12885-015-1127-y

47. Kuang X, Zhu J, Peng Z, Wang J, Chen Z. Transducin (Beta)-Like 1 X-Linked Receptor 1 Correlates with Clinical Prognosis and Epithelial-Mesenchymal Transition in Hepatocellular Carcinoma. *Dig Dis Sci* (2016) **61**:489–500. doi:10.1007/s10620-015-3879-2

48. Li L, Xu L, Yan J, Zhen Z-J, Ji Y, Liu C-Q, Lau WY, Zheng L, Xu J. CXCR2-CXCL1 axis is correlated with neutrophil infiltration and predicts a poor prognosis in hepatocellular carcinoma. *J Exp Clin Cancer Res CR* (2015) **34**:129. doi:10.1186/s13046-015-0247-1

49. Huang W-J, Jeng Y-M, Lai H-S, Fong I-U, Sheu F-YB, Lai P-L, Yuan R-H. Expression of hypoxic marker carbonic anhydrase IX predicts poor prognosis in resectable hepatocellular carcinoma. *PloS One* (2015) **10**:e0119181. doi:10.1371/journal.pone.0119181

50. Kim B-Y, Choi DW, Woo SR, Park E-R, Lee J-G, Kim S-H, Koo I, Park S-H, Han CJ, Kim SB, et al. Recurrence-associated pathways in hepatitis B virus-positive hepatocellular carcinoma. *BMC Genomics* (2015) **16**:279. doi:10.1186/s12864-015-1472-x

51. Wang W, Li G-Y, Zhu J-Y, Huang D-B, Zhou H-C, Zhong W, Ji C-S. Overexpression of AGGF1 is correlated with angiogenesis and poor prognosis of hepatocellular carcinoma. *Med Oncol Northwood Lond Engl* (2015) **32**:131. doi:10.1007/s12032-015-0574-2

52. Tang B, Tang F, Li B, Yuan S, Xu Q, Tomlinson S, Jin J, Hu W, He S. High USP22 expression indicates poor prognosis in hepatocellular carcinoma. *Oncotarget* (2015) **6**:12654–12667. doi:10.18632/oncotarget.3705

53. Chen H-W, Qiao H-Y, Li H-C, Li Z-F, Zhang H-J, Pei L, Liu H-W, Jin L, Wang D, Li J-L. Prognostic significance of Nemo-like kinase expression in patients with hepatocellular carcinoma. *Tumour Biol J Int Soc Oncodevelopmental Biol Med* (2015) **36**:8447–8453. doi:10.1007/s13277-015-3609-6

54. Chen H, Miao J, Li H, Wang C, Li J, Zhu Y, Wang J, Wu X, Qiao H. Expression and prognostic significance of p21-activated kinase 6 in hepatocellular carcinoma. *J Surg Res* (2014) **189**:81–88. doi:10.1016/j.jss.2014.01.049

55. Murakami K, Kasajima A, Kawagishi N, Sekiguchi S, Fujishima F, Watanabe M, Sato Y, Ohuchi N, Sasano H. The prognostic significance of vasohibin 1-associated angiogenesis in patients with hepatocellular carcinoma. *Hum Pathol* (2014) **45**:589–597. doi:10.1016/j.humpath.2013.10.028

56. Lv Y, Wang W, Jia WD, Sun QK, Huang M, Zhou HC, Xia HH, Liu WB, Chen H, Sun SN, et al. High preoparative levels of serum periostin are associated with poor prognosis in patients with hepatocellular carcinoma after hepatectomy. *Eur J Surg Oncol J Eur Soc Surg Oncol Br Assoc Surg Oncol* (2013) **39**:1129–1135. doi:10.1016/j.ejso.2013.06.023

57. Zhou L, Rui J-A, Wang S-B, Chen S-G, Qu Q. Prognostic factors of solitary large hepatocellular carcinoma: the importance of differentiation grade. *Eur J Surg Oncol J Eur Soc Surg Oncol Br Assoc Surg Oncol* (2011) **37**:521–525. doi:10.1016/j.ejso.2011.03.137

58. Lee YJ, Hah YJ, Ha YJ, Kang YN, Kang KJ, Hwang JS, Chung WJ, Cho KB, Park KS, Kim ES, et al. The autophagy-related marker LC3 can predict prognosis in human hepatocellular carcinoma. *PloS One* (2013) **8**:e81540. doi:10.1371/journal.pone.0081540

59. Tu K, Yang W, Li C, Zheng X, Lu Z, Guo C, Yao Y, Liu Q. Fbxw7 is an independent prognostic marker and induces apoptosis and growth arrest by regulating YAP abundance in hepatocellular carcinoma. *Mol Cancer* (2014) **13**:110. doi:10.1186/1476-4598-13-110

60. Chen H-W, Huang X-D, Li H-C, He S, Ni R-Z, Chen C-H, Peng C, Wu G, Wang G-H, Wang Y-Y, et al. Expression of FOXJ1 in hepatocellular carcinoma: correlation with patients’ prognosis and tumor cell proliferation. *Mol Carcinog* (2013) **52**:647–659. doi:10.1002/mc.21904

61. Zhao Y-M, Zhou J-M, Wang L-R, He H-W, Wang X-L, Tao Z-H, Sun H-C, Wu W-Z, Fan J, Tang Z-Y, et al. HIWI is associated with prognosis in patients with hepatocellular carcinoma after curative resection. *Cancer* (2012) **118**:2708–2717. doi:10.1002/cncr.26524

62. Zhang G, Liu T, Wang Z. Downregulation of MAGI1 associates with poor prognosis of hepatocellular carcinoma. *J Investig Surg Off J Acad Surg Res* (2012) **25**:93–99. doi:10.3109/08941939.2011.606875

63. Hu S, Wu X, Zhou B, Xu Z, Qin J, Lu H, Lv L, Gao Y, Deng L, Yin J, et al. IMP3 combined with CD44s, a novel predictor for prognosis of patients with hepatocellular carcinoma. *J Cancer Res Clin Oncol* (2014) **140**:883–893. doi:10.1007/s00432-014-1639-x

64. Wang Y, Yu Y-N, Song S, Li T-J, Xiang J-Y, Zhang H, Lu M-D, Ji F, Hu L-Q. JAB1 and phospho-Ser10 p27 expression profile determine human hepatocellular carcinoma prognosis. *J Cancer Res Clin Oncol* (2014) **140**:969–978. doi:10.1007/s00432-014-1646-y

65. Liu J, Ni W, Xiao M, Jiang F, Ni R. Decreased expression and prognostic role of mitogen-activated protein kinase phosphatase 4 in hepatocellular carcinoma. *J Gastrointest Surg Off J Soc Surg Aliment Tract* (2013) **17**:756–765. doi:10.1007/s11605-013-2138-0

66. Lv Y, Wang W, Jia W-D, Sun Q-K, Li J-S, Ma J-L, Liu W-B, Zhou H-C, Ge Y-S, Yu J-H, et al. High-level expression of periostin is closely related to metastatic potential and poor prognosis of hepatocellular carcinoma. *Med Oncol Northwood Lond Engl* (2013) **30**:385. doi:10.1007/s12032-012-0385-7

67. Guo X, Xiong L, Zou L, Zhao J. Upregulation of bone morphogenetic protein 4 is associated with poor prognosis in patients with hepatocellular carcinoma. *Pathol Oncol Res POR* (2012) **18**:635–640. doi:10.1007/s12253-011-9488-2

68. Chen H-C, Jeng Y-M, Yuan R-H, Hsu H-C, Chen Y-L. SIRT1 promotes tumorigenesis and resistance to chemotherapy in hepatocellular carcinoma and its expression predicts poor prognosis. *Ann Surg Oncol* (2012) **19**:2011–2019. doi:10.1245/s10434-011-2159-4

69. Chen L, Jiang M, Yuan W, Tang H. miR-17-5p as a novel prognostic marker for hepatocellular carcinoma. *J Investig Surg Off J Acad Surg Res* (2012) **25**:156–161. doi:10.3109/08941939.2011.618523

70. Park Y, Yu E. Expression of metallothionein-1 and metallothionein-2 as a prognostic marker in hepatocellular carcinoma. *J Gastroenterol Hepatol* (2013) **28**:1565–1572. doi:10.1111/jgh.12261

71. Noda T, Yamamoto H, Takemasa I, Yamada D, Uemura M, Wada H, Kobayashi S, Marubashi S, Eguchi H, Tanemura M, et al. PLOD2 induced under hypoxia is a novel prognostic factor for hepatocellular carcinoma after curative resection. *Liver Int Off J Int Assoc Study Liver* (2012) **32**:110–118. doi:10.1111/j.1478-3231.2011.02619.x

72. Zhang J, Tu K, Yang W, Li C, Yao Y, Zheng X, Liu Q. Evaluation of Jagged2 and Gli1 expression and their correlation with prognosis in human hepatocellular carcinoma. *Mol Med Rep* (2014) **10**:749–754. doi:10.3892/mmr.2014.2246

73. Shindoh J, Andreou A, Aloia TA, Zimmitti G, Lauwers GY, Laurent A, Nagorney DM, Belghiti J, Cherqui D, Poon RT-P, et al. Microvascular invasion does not predict long-term survival in hepatocellular carcinoma up to 2 cm: reappraisal of the staging system for solitary tumors. *Ann Surg Oncol* (2013) **20**:1223–1229. doi:10.1245/s10434-012-2739-y

74. Huang G-L, Li B-K, Zhang M-Y, Wei R-R, Yuan Y-F, Shi M, Chen X-Q, Huang L, Zhang H-Z, Liu W, et al. Allele loss and down-regulation of heparanase gene are associated with the progression and poor prognosis of hepatocellular carcinoma. *PloS One* (2012) **7**:e44061. doi:10.1371/journal.pone.0044061

75. Yuan R-H, Chang K-T, Chen Y-L, Hsu H-C, Lee P-H, Lai P-L, Jeng Y-M. S100P expression is a novel prognostic factor in hepatocellular carcinoma and predicts survival in patients with high tumor stage or early recurrent tumors. *PloS One* (2013) **8**:e65501. doi:10.1371/journal.pone.0065501

76. Zhou L, Rui J-A, Wang S-B, Chen S-G, Qu Q. Risk factors of poor prognosis and portal vein tumor thrombosis after curative resection of solitary hepatocellular carcinoma. *Hepatobiliary Pancreat Dis Int HBPD INT* (2013) **12**:68–73.

77. Chen Y-L, Chen C-H, Hu R-H, Ho M-C, Jeng Y-M. Elevated preoperative serum CA19-9 levels in patients with hepatocellular carcinoma is associated with poor prognosis after resection. *ScientificWorldJournal* (2013) **2013**:380797. doi:10.1155/2013/380797

78. Han DH, Choi GH, Kim KS, Choi JS, Park YN, Kim SU, Park JY, Ahn SH, Han K-H. Prognostic significance of the worst grade in hepatocellular carcinoma with heterogeneous histologic grades of differentiation. *J Gastroenterol Hepatol* (2013) **28**:1384–1390. doi:10.1111/jgh.12200

79. Xu J, Liu C, Zhou L, Tian F, Tai M-H, Wei J-C, Qu K, Meng F-D, Zhang L-Q, Wang Z-X, et al. Distinctions between clinicopathological factors and prognosis of alpha-fetoprotein negative and positive hepatocelluar carcinoma patients. *Asian Pac J Cancer Prev APJCP* (2012) **13**:559–562.

80. Qiu D-M, Wang G-L, Chen L, Xu Y-Y, He S, Cao X-L, Qin J, Zhou J-M, Zhang Y-X, E Q. The expression of beclin-1, an autophagic gene, in hepatocellular carcinoma associated with clinical pathological and prognostic significance. *BMC Cancer* (2014) **14**:327. doi:10.1186/1471-2407-14-327

81. Hu B, Xiong Y, Ni R, Wei L, Jiang D, Wang G, Wu D, Xu T, Zhao F, Zhu M, et al. The downregulation of ErbB3 binding protein 1 (EBP1) is associated with poor prognosis and enhanced cell proliferation in hepatocellular carcinoma. *Mol Cell Biochem* (2014) **396**:175–185. doi:10.1007/s11010-014-2153-9

82. Zhu J-Y, Sun Q-K, Wang W, Jia W-D. High-level expression of HOXB13 is closely associated with tumor angiogenesis and poor prognosis of hepatocellular carcinoma. *Int J Clin Exp Pathol* (2014) **7**:2925–2933.

83. Kim JM, Kwon CHD, Joh J-W, Park JB, Lee JH, Kim SJ, Paik SW, Park CK, Yoo BC. Outcomes after curative hepatectomy in patients with non-B non-C hepatocellular carcinoma and hepatitis B virus hepatocellular carcinoma from non-cirrhotic liver. *J Surg Oncol* (2014) **110**:976–981. doi:10.1002/jso.23772

84. Liu W, Xu G, Jia W, Li J, Ma J, Chen K, Wang Z, Ge Y, Ren W, Yu J, et al. Prognostic significance and mechanisms of patterned matrix vasculogenic mimicry in hepatocellular carcinoma. *Med Oncol Northwood Lond Engl* (2011) **28 Suppl 1**:S228-238. doi:10.1007/s12032-010-9706-x

85. Shin E, Ryu HS, Kim S-H, Jung H, Jang J-J, Lee K. The clinicopathological significance of heat shock protein 70 and glutamine synthetase expression in hepatocellular carcinoma. *J Hepato-Biliary-Pancreat Sci* (2011) **18**:544–550. doi:10.1007/s00534-010-0367-0

86. Yuan R-H, Lai H-S, Hsu H-C, Lai P-L, Jeng Y-M. Expression of bile duct transcription factor HNF1β predicts early tumor recurrence and is a stage-independent prognostic factor in hepatocellular carcinoma. *J Gastrointest Surg Off J Soc Surg Aliment Tract* (2014) **18**:1784–1794. doi:10.1007/s11605-014-2596-z

87. Zhou L, Rui J-A, Wang S-B, Chen S-G, Qu Q. Clinicopathological predictors of poor survival and recurrence after curative resection in hepatocellular carcinoma without portal vein tumor thrombosis. *Pathol Oncol Res POR* (2015) **21**:131–138. doi:10.1007/s12253-014-9798-2

88. Guo X, Xiong L, Yu L, Li R, Wang Z, Ren B, Dong J, Li B, Wang D. Increased level of nucleolin confers to aggressive tumor progression and poor prognosis in patients with hepatocellular carcinoma after hepatectomy. *Diagn Pathol* (2014) **9**:175. doi:10.1186/s13000-014-0175-y

89. Chang R, Wei L, Lu Y, Cui X, Lu C, Liu L, Jiang D, Xiong Y, Wang G, Wan C, et al. Upregulated expression of ubiquitin-conjugating enzyme E2Q1 (UBE2Q1) is associated with enhanced cell proliferation and poor prognosis in human hapatocellular carcinoma. *J Mol Histol* (2015) **46**:45–56. doi:10.1007/s10735-014-9596-x

90. Zhang X-F, Pan Q-Z, Pan K, Weng D-S, Wang Q-J, Zhao J-J, He J, Liu Q, Wang D-D, Jiang S-S, et al. Expression and prognostic role of ubiquitination factor E4B in primary hepatocellular carcinoma. *Mol Carcinog* (2016) **55**:64–76. doi:10.1002/mc.22259

91. Yang L, Hu B, Zhang Y, Qiang S, Cai J, Huang W, Gong C, Zhang T, Zhang S, Xu P, et al. Suppression of the nuclear transporter-KPNβ1 expression inhibits tumor proliferation in hepatocellular carcinoma. *Med Oncol Northwood Lond Engl* (2015) **32**:128. doi:10.1007/s12032-015-0559-1

92. Ha SY, Song DH, Hwang SH, Cho SY, Park C-K. Expression of prothymosin alpha predicts early recurrence and poor prognosis of hepatocellular carcinoma. *Hepatobiliary Pancreat Dis Int HBPD INT* (2015) **14**:171–177.

93. Ha SY, Sohn I, Hwang SH, Yang JW, Park C-K. The prognosis of hepatocellular carcinoma after curative hepatectomy in young patients. *Oncotarget* (2015) **6**:18664–18673. doi:10.18632/oncotarget.4330

94. Cong X, Lu C, Huang X, Yang D, Cui X, Cai J, Lv L, He S, Zhang Y, Ni R. Increased expression of glycinamide ribonucleotide transformylase is associated with a poor prognosis in hepatocellular carcinoma, and it promotes liver cancer cell proliferation. *Hum Pathol* (2014) **45**:1370–1378. doi:10.1016/j.humpath.2013.11.021

95. Pedica F, Ruzzenente A, Bagante F, Capelli P, Cataldo I, Pedron S, Iacono C, Chilosi M, Scarpa A, Brunelli M, et al. A re-emerging marker for prognosis in hepatocellular carcinoma: the add-value of fishing c-myc gene for early relapse. *PloS One* (2013) **8**:e68203. doi:10.1371/journal.pone.0068203

96. Guo X, Xiong L, Sun T, Peng R, Zou L, Zhu H, Zhang J, Li H, Zhao J. Expression features of SOX9 associate with tumor progression and poor prognosis of hepatocellular carcinoma. *Diagn Pathol* (2012) **7**:44. doi:10.1186/1746-1596-7-44

97. Chen P, Zhao X, Ma L. Downregulation of microRNA-100 correlates with tumor progression and poor prognosis in hepatocellular carcinoma. *Mol Cell Biochem* (2013) **383**:49–58. doi:10.1007/s11010-013-1753-0

98. Mizuno H, Honda M, Shirasaki T, Yamashita T, Yamashita T, Mizukoshi E, Kaneko S. Heterogeneous nuclear ribonucleoprotein A2/B1 in association with hTERT is a potential biomarker for hepatocellular carcinoma. *Liver Int Off J Int Assoc Study Liver* (2012) **32**:1146–1155. doi:10.1111/j.1478-3231.2012.02778.x

99. Pan H-Z, Dong A-B, Wang L, Tan S-S, Yang Q, Tong X-Y, Liang J, Wang J-R. Significance of relaxin-2 expression in hepatocellular carcinoma: relation with clinicopathological parameters. *Eur Rev Med Pharmacol Sci* (2013) **17**:1095–1101.

100. Wu D-H, Jia C-C, Chen J, Lin Z-X, Ruan D-Y, Li X, Lin Q, Min-Dong null, Ma X-K, Wan X-B, et al. Autophagic LC3B overexpression correlates with malignant progression and predicts a poor prognosis in hepatocellular carcinoma. *Tumour Biol J Int Soc Oncodevelopmental Biol Med* (2014) **35**:12225–12233. doi:10.1007/s13277-014-2531-7

101. Sasaki K, Matsuda M, Ohkura Y, Kawamura Y, Inoue M, Hashimoto M, Ikeda K, Kumada H, Watanabe G. The influence of histological differentiation grade on the outcome of liver resection for hepatocellular carcinomas 2 cm or smaller in size. *World J Surg* (2015) **39**:1134–1141. doi:10.1007/s00268-014-2806-6

102. Sasaki K, Matsuda M, Ohkura Y, Kawamura Y, Inoue M, Hashimoto M, Ikeda K, Kumada H, Watanabe G. In hepatocellular carcinomas, any proportion of poorly differentiated components is associated with poor prognosis after hepatectomy. *World J Surg* (2014) **38**:1147–1153. doi:10.1007/s00268-013-2374-1

103. Lu J-W, Chang J-G, Yeh K-T, Chen R-M, Tsai JJP, Su W-W, Hu R-M. Increased expression of PRL-1 protein correlates with shortened patient survival in human hepatocellular carcinoma. *Clin Transl Oncol Off Publ Fed Span Oncol Soc Natl Cancer Inst Mex* (2012) **14**:287–293. doi:10.1007/s12094-012-0797-z

104. Geng M, Cao Y-C, Chen Y-J, Jiang H, Bi L-Q, Liu X-H. Loss of Wnt5a and Ror2 protein in hepatocellular carcinoma associated with poor prognosis. *World J Gastroenterol* (2012) **18**:1328–1338. doi:10.3748/wjg.v18.i12.1328

105. Chao J, Zhang X-F, Pan Q-Z, Zhao J-J, Jiang S-S, Wang Y, Zhang J-H, Xia J-C. Decreased expression of TRIM3 is associated with poor prognosis in patients with primary hepatocellular carcinoma. *Med Oncol Northwood Lond Engl* (2014) **31**:102. doi:10.1007/s12032-014-0102-9

106. Liu A-W, Cai J, Zhao X-L, Jiang T-H, He T-F, Fu H-Q, Zhu M-H, Zhang S-H. ShRNA-targeted MAP4K4 inhibits hepatocellular carcinoma growth. *Clin Cancer Res Off J Am Assoc Cancer Res* (2011) **17**:710–720. doi:10.1158/1078-0432.CCR-10-0331

107. Lu J-W, Chang J-G, Yeh K-T, Chen R-M, Tsai JJP, Hu R-M. Decreased expression of p39 is associated with a poor prognosis in human hepatocellular carcinoma. *Med Oncol Northwood Lond Engl* (2011) **28 Suppl 1**:S239-245. doi:10.1007/s12032-010-9707-9

108. Luo H, Dong Z, Zou J, Zeng Q, Wu D, Liu L. Down-regulation of RhoE is associated with progression and poor prognosis in hepatocellular carcinoma. *J Surg Oncol* (2012) **105**:699–704. doi:10.1002/jso.23019

109. Jia S-W, Fu S, Wang F, Shao Q, Huang H-B, Shao J-Y. ALK gene copy number gain and its clinical significance in hepatocellular carcinoma. *World J Gastroenterol* (2014) **20**:183–192. doi:10.3748/wjg.v20.i1.183

110. Harimoto N, Shirabe K, Ikegami T, Yoshizumi T, Maeda T, Kajiyama K, Yamanaka T, Maehara Y. Postoperative complications are predictive of poor prognosis in hepatocellular carcinoma. *J Surg Res* (2015) **199**:470–477. doi:10.1016/j.jss.2015.06.012

111. Zhang Y, Li Y, Lin C, Ding J, Liao G, Tang B. Aberrant upregulation of 14-3-3σ and EZH2 expression serves as an inferior prognostic biomarker for hepatocellular carcinoma. *PloS One* (2014) **9**:e107251. doi:10.1371/journal.pone.0107251

112. Huang Y-H, Chen Z-K, Huang K-T, Li P, He B, Guo X, Zhong J-Q, Zhang Q-Y, Shi H-Q, Song Q-T, et al. Decreased expression of LKB1 correlates with poor prognosis in hepatocellular carcinoma patients undergoing hepatectomy. *Asian Pac J Cancer Prev APJCP* (2013) **14**:1985–1988.

113. Cao X, Xia Y, Yang J, Jiang J, Chen L, Ni R, Li L, Gu Z. Clinical and biological significance of never in mitosis gene A-related kinase 6 (NEK6) expression in hepatic cell cancer. *Pathol Oncol Res POR* (2012) **18**:201–207. doi:10.1007/s12253-011-9429-0

114. Wang Y, Ye Z, Meng X-Q, Zheng S-S. Expression of HLA-G in patients with hepatocellular carcinoma. *Hepatobiliary Pancreat Dis Int HBPD INT* (2011) **10**:158–163.
